# A Small‐Molecule Drug for the Self‐Checking of Mitophagy

**DOI:** 10.1002/anie.202421269

**Published:** 2025-01-22

**Authors:** Yanan Gao, Qingjie Bai, Youxiao Ren, Xintian Shao, Mengrui Zhang, Luling Wu, Simon E. Lewis, Tony D. James, Xiaoyuan Chen, Qixin Chen

**Affiliations:** ^1^ State Key Laboratory of Advanced Drug Delivery and Release Systems School of Pharmaceutical Sciences Neck-Shoulder and Lumbocrural Pain Hospital Medical Science and Technology Innovation Center Shandong First Medical University & Shandong Academy of Medical Sciences Jinan Shandong 250117 PR China; ^2^ Departments of Diagnostic Radiology Chemical and Biomolecular Engineering and Biomedical Engineering Yong Loo Lin School of Medicine and College of Design and Engineering National University of Singapore Singapore 119074 Singapore; ^3^ Department of Chemistry University of Bath Bath BA2 7AY U.K.; ^4^ School of Chemistry and Chemical Engineering Henan Normal University Xinxiang 453007 People's Republic of China; ^5^ Clinical Imaging Research Centre Centre for Translational Medicine Yong Loo Lin School of Medicine National University of Singapore Singapore 117599 Singapore; ^6^ Nanomedicine Translational Research Program Yong Loo Lin School of Medicine National University of Singapore Singapore 117597 Singapore; ^7^ Theranostics Center of Excellence (TCE) Yong Loo Lin School of Medicine National University of Singapore 11 Biopolis Way, Helios Singapore 138667; ^8^ Institute of Molecular and Cell Biology Agency for Science Technology and Research (A*STAR) 61 Biopolis Drive, Proteos Singapore 138673 Singapore; ^9^ Department of Pharmacy and Pharmaceutical Sciences National University of Singapore Lower Kent Ridge Road, 4 Science Drive 2 117544 Singapore

**Keywords:** Mitochondria, Lysosomes, Subcellular dynamics, Dual localization, Self-checking drug

## Abstract

Mitophagy, particularly in the context of drugs that disrupt mitochondrial membrane potential (MMP), represents a critical focus in pharmacology. However, the discovery and evaluation of MMP‐disrupting drugs are often hampered using commercially available marker molecules that target similar or identical zones. These markers can significantly interfere with, obscure, or amplify the functional effects of MMP‐targeting drugs, frequently leading to clinical failures. In response to this challenge, we propose a “one‐two punch” drug design strategy that integrates both target‐zone drug functionality and non‐target zone biological reporting within a single small‐molecule drug. We have developed a novel proof‐of‐concept mitophagy self‐check drug (**MitoSC**) that exhibits dual‐color and dual‐localization properties. The functional component of this system is a variable **MitoSC** that disrupts mitochondrial membrane potential (MMP) homeostasis, thereby inducing mitophagy. Upon activation, this component transforms into a blue‐fluorescent monomer (**MitoSC‐fun**) specifically within the mitochondrial target zone. Concurrently, the biological reporting component is represented by a red‐fluorescent monomer (**MitoSC‐rep**) that localizes to lysosomes, the non‐target zone. As mitophagy progresses, the fluorescent signals from **MitoSC‐rep** (lysosomes) and **MitoSC‐fun** (mitochondria) converge, enabling real‐time monitoring of the mitophagic process. This strategy combines potent drug functionality with robust biological reporting, thereby minimizing interference and eliminating the complexities associated with external detection. Our findings underscore the potential of a single‐molecule drug to exert target‐zone specific actions while simultaneously providing non‐target zone self‐checking, offering a new perspective for drug design.

## Graphical abstract

This work introduces a innovative “one‐two punch” drug design strategy, exemplified by the mitophagy self‐check drug. This strategy integrates both target‐zone drug functionality and non‐target zone biological reporting into a single small‐molecule drug, effectively addressing the challenges associated with conventional marker interference in drug evaluation.

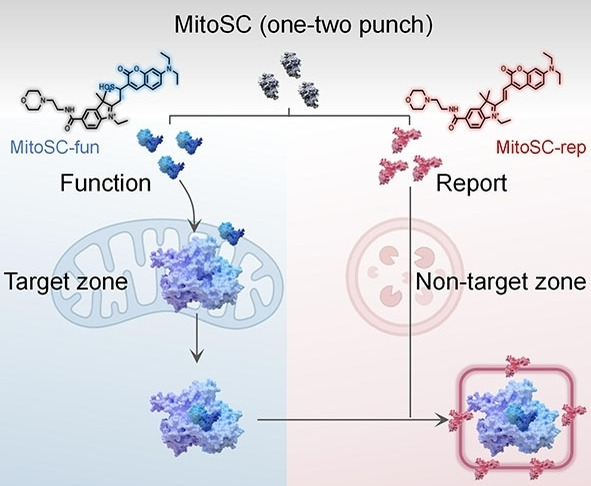

## Introduction

Mitophagy is not only a mechanism for the removal and renewal of damaged mitochondria but also plays a crucial role in intracellular material recycling. Given the strong association between mitophagy and the pathogenesis of numerous clinical diseases, there has been a significant increase in efforts to develop drugs that induce mitophagy by disrupting the mitochondrial membrane potential (MMP), with the aim of precisely controlling the progression of related diseases.[^1^, ^2^, ^3^, ^4^] For example, Mito‐LND^[5]^ and AT‐101^[6]^ achieve their anticancer effects by disrupting the MMP, thereby impacting mitochondria function and activating autophagy‐related pathways. However, despite the discovery and development of these promising drugs, a gap remains between the current status and optimal clinical outcomes.

Notably, the search for drug molecules that can simultaneously function and self‐check has been a strong focus in the fields of medicinal and analytical chemistry.[^7^, ^8^, ^9^] Conventional functional assessments typically rely on exogenous probes, such as fluorescent labels.[^10^, ^11^, ^12^] These commercial labels may infiltrate target sites to compete with drugs for the same targets, leading to significant interference, masking, or exaggeration of drug effects, This can result in false positives and uncertain clinical efficacy.[^13^, ^14^, ^15^] Moreover, current drug design strategies primarily focus on functional optimization, aiming to achieve optimal therapeutic outcomes through targeted modifications.[^16^, ^17^] While this approach is effective for chemically synthesized drugs with well‐defined targets, it presents challenges in functional validation.[^18^, ^19^] Ideally, non‐competitive molecules capable of directly detecting functional changes after drug‐target binding would be preferable, However, limitations in understanding drug and target activities during early screening and discovery often render this approach unfeasible.[^20^, ^21^, ^22^] Even for drugs with known targets, using non‐competitive probes necessitates multi‐step staining procedures, potentially exposing cells to adverse conditions and increasing experimental complexity.[^23^, ^24^] To date, no drug design paradigm has successfully integrated functional optimization with biological reporting.

Despite the prevalence of the therapeutic‐diagnostic probe/drug design strategy in emerging drug integration, a consistent issue persists: the functional and reporting doses for drugs targeting the same region often coincide, leading to conflicts in their respective dosage requirements.[^25^, ^26^, ^27^] The inability to effectively decouple target‐zone functionality from biological reporting in therapeutic‐diagnostic drugs, particularly toxic anticancer agents, presents a significant bottleneck that hinders their clinical adoption.[^28^, ^29^]

**Figure 1:**
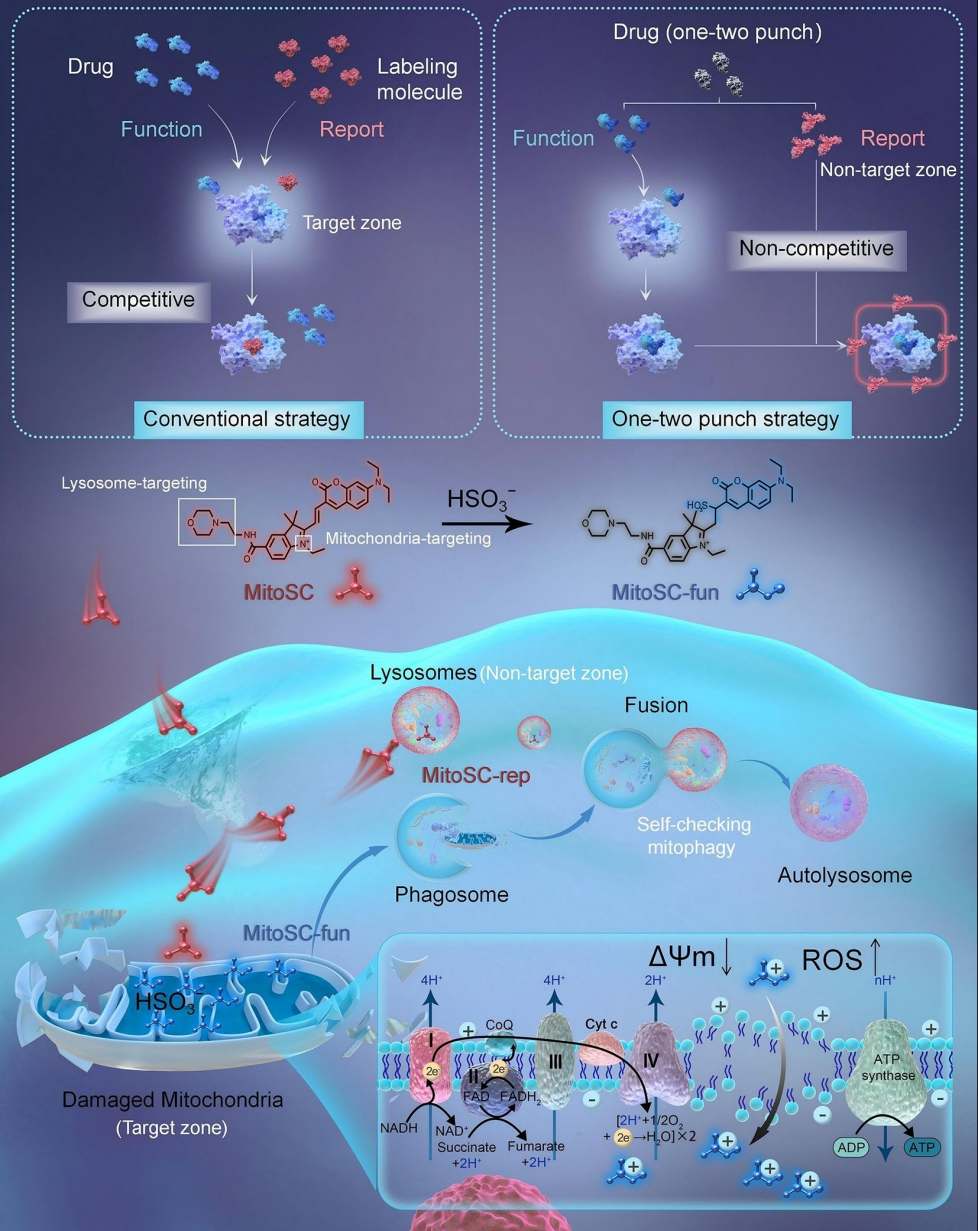
Design of one‐two punch drug (**MitoSC**) for self‐checking mitophagy. Conventional drug functional assessment strategies often rely on fluorescent‐labelling molecules, which may compete with drugs for the same targets. This competition can interfere with, mask, or exaggerate drug effects, leading to false positives and uncertain clinical efficacy. To address this issue, we propose a novel drug design strategy termed “one‐two punch”, which enables a single drug to exhibit different localizations – one responsible for upstream target‐zone function and the other for downstream non‐target‐zone biological reporting – without competing for the drug target. Using the mitochondrial membrane potential (MMP) as an example, we designed a self‐checking mitophagy drug **MitoSC** with dual‐color and dual‐localization properties. Upon entering the cell, **MitoSC** selectively targets both the mitochondria and lysosomes. In the mitochondria, it disrupts MMP homeostasis, leading to mitochondrial damage, initiation of mitophagy and conversion into **MitoSC‐fun**. Simultaneously, **MitoSC‐rep** in lysosomes, reports mitophagy in real‐time through color merging. **MitoSC** (**MitoSC‐rep**) transforms into **MitoSC‐fun** after entering the mitochondria, and the chemical structures of **MitoSC** and **MitoSC‐rep** are completely identical.

To address these challenges, we introduce a novel drug design strategy termed “one‐two punch”, which envisions a single drug molecule capable of both upstream target‐zone functionality and downstream non‐target‐zone biological reporting. Using MMP[^30^, ^31^, ^32^, ^33^] as a model, we developed **MitoSC**, a mitophagy self‐checking drug with dual‐color and dual‐localization properties. **MitoSC** exists in two intracellular forms: **MitoSC‐rep**, a non‐target zone reporter localized in lysosomes, that emits red fluorescence, while **MitoSC‐fun**, a transformable molecule in the mitochondria, emits blue fluorescence. Upon disrupting MMP homeostasis and initiating mitophagy, **MitoSC** is converted into **MitoSC‐fun**. Concurrently, **MitoSC‐rep** within the lysosomes reports mitophagy through color merging. This integrated approach eliminates the need for exogenous fluorescent probes, thereby avoiding target competition and redundant labeling processes inherent in current drug design frameworks. By fusing upstream target‐zone functionality with downstream non‐target‐zone detection, we achieve a paradigm shift for integrating functionality and biological reporting using a single drug molecule (Figure 1).

## Results and Discussion

### Design and Synthesis of “One‐Two Punch” Drug (MitoSC) for Self‐Checking Mitophagy

Drawing inspiration from the reported activation of mitophagy following MMP reduction‐induced mitochondrial damage, we developed a small‐molecule drug, **MitoSC**, which integrates upstream functionality with downstream biological reporting within a single entity. The design principles are as follows. First, for the **MitoSC** functional moiety, which targets the MMP, we designed a nitrogen cation^[34]^ to accumulate within the mitochondrial inner membrane, thereby disrupting proton homeostasis and destabilizing the MMP. Second, to prevent competition with the target‐zone of **MitoSC**, **MitoSC** reporter moiety selectively localized to the lysosomes, where lysosomal–mitochondrial fusion can then signal mitophagy. Third, dual localization was achieved within a single molecule: the nitrogen cation localizes in the mitochondria, whereas the morpholine group^[35]^ localizes in the lysosomes. Finally, dual‐color emission was observed: coumarin emits ~500 nm (blue),^[13]^ while hemicyanine emits ~650 nm (red),[^36^, ^37^] enabling visualization of the target‐zone functionality (mitochondria) and non‐target‐zone reporting (lysosomes).

Upon cellular entry, **MitoSC** was partitioned: some molecules were sequestered by lysosomes as **MitoSC‐rep**, emitting red fluorescence (~650 nm), while others, driven by high MMP in tumor cells,^[38]^ localized to the mitochondria. In the slightly alkaline mitochondrial environment (pH=8.0), **MitoSC** transformed into **MitoSC‐fun** upon interaction with reactive sulfur species (RSSs) such as H_2_S and SO_2_ (Figure S1–2), resulting in the release of blue fluorescence (~500 nm) from coumarin.[^39^, ^40^, ^41^, ^42^] **MitoSC** disrupts the MMP, causing mitochondrial damage and initiating mitophagy. Concurrently, **MitoSC‐rep** in the lysosomes fuses with **MitoSC‐fun**‐damaged mitochondria, thereby reporting mitophagy through dual‐color fluorescence. Based on these design concepts and with 5‐carboxy‐1‐ethyl‐2,3,3‐trimethyl‐3H‐indol‐1‐ium iodide, 7‐(diethylamino)‐2‐oxo‐2H‐chromene‐3‐carbaldehyde and 4‐(2‐aminoethyl) morpholine as starting materials, we successfully synthesized **MitoSC** using straightforward steps (Figure S3–4). Its chemical structure was confirmed by HR‐MS, ^1^H NMR, and ^13^C NMR, etc. (Figure S5–17).

In summary, we synthesized **MitoSC**, a single molecule that transitions into two distinct visual reporters within different organelles. **MitoSC** operates within the mitochondria and transforms into **MitoSC‐fun** (blue), while **MitoSC‐rep** (red) reports from the lysosomes. This color‐overlap approach self‐checking of the progress of mitophagy, obviates the need for exogenous detectors or multiple labeling steps.

### MitoSC Enables Dual Localization/Color within the Mitochondria and Lysosomes

**Figure 2:**
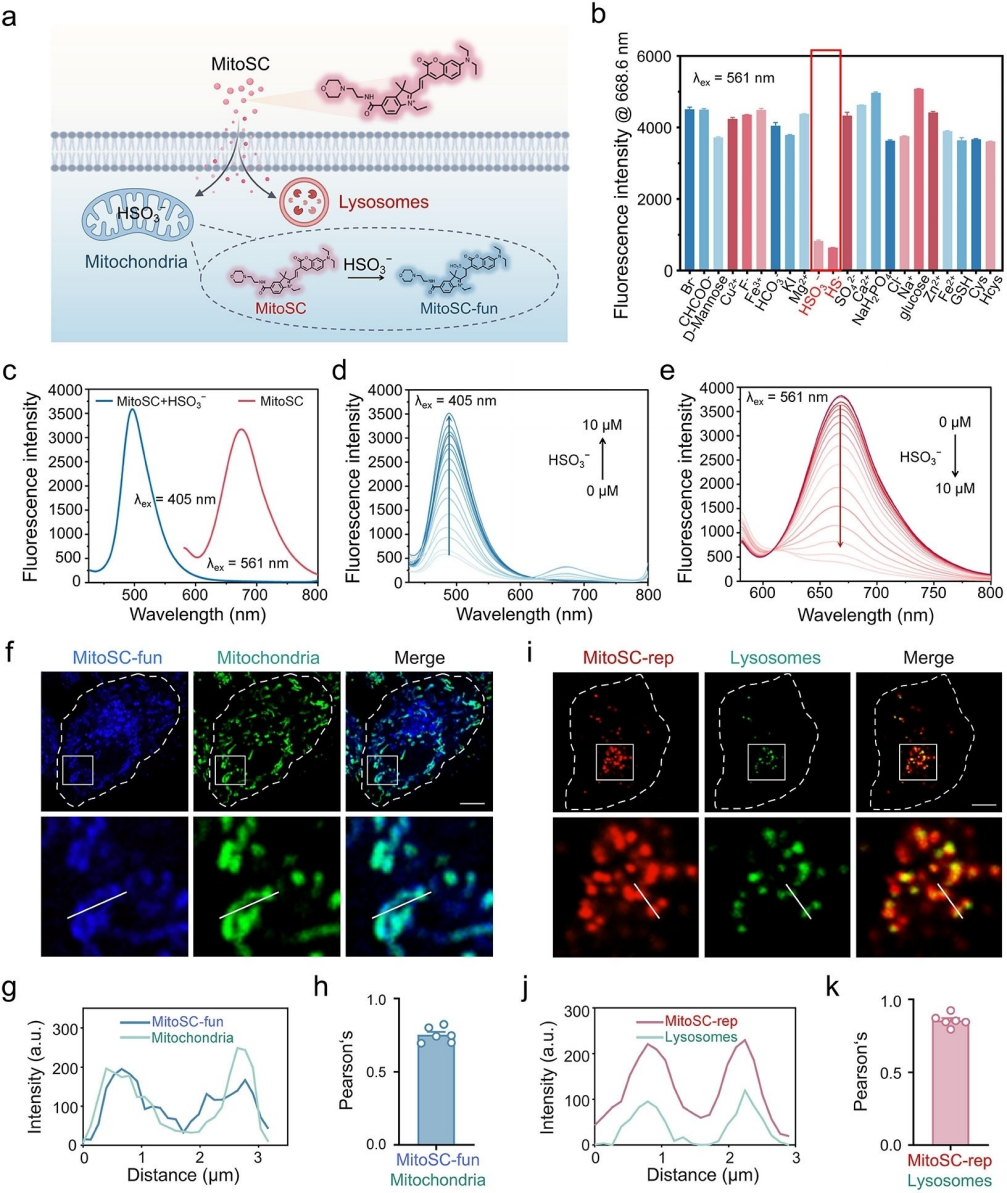
**MitoSC** enables dual‐localization/color visualization within the mitochondria and lysosomes. (a) Schematic diagram illustrating **MitoSC** labeling of the mitochondria and lysosomes. Created in BioRender. Shao, S. (2024) https://biorender.com/h43h388. (b) The fluorescence intensity at 668.6 nm of **MitoSC** in PBS solutions (pH=7.4) at room temperature after the addition of Ca^2+^, HSO_3_^−^, GSH and various related substances (10 μM), (λ_ex_=561 nm, ex slit: 10 nm, em slit: 10 nm) (*n*=3). (c) Fluorescence spectra of **MitoSC** (10 μM) with or without HSO_3_^−^ (10 μM) in PBS solutions (pH=7.4) at room temperature (λ_ex_=405 nm, ex slit: 10 nm, em slit: 10 nm; λ_ex_=561 nm, ex slit: 10 nm, em slit: 10 nm). (d) Fluorescence spectra of **MitoSC** in response to different concentrations of HSO_3_^−^ (0–10 μM) (λ_ex_=405 nm, ex slit: 10 nm, em slit: 10 nm). (e) Fluorescence spectra of **MitoSC** in response to different concentrations of HSO_3_^−^ (0–10 μM) (λ_ex_=561 nm, ex slit: 10 nm, em slit: 10 nm). (f) Confocal imaging of HeLa cells co‐stained with **MitoSC** (10 μM) and commercial mitochondrial dye (MitoTracker™ Green FM, MTG); the white rectangle is enlarged (scale bar=5 μm). (g) Fluorescence intensity distribution of **MitoSC‐fun** and mitochodria at the position indicated by the white solid line in (f). (h) The Pearson correlation coefficient for **MitoSC‐fun** with mitochondria was 0.75 (*n*=6). (i) Confocal imaging of HeLa cells co‐stained with **MitosSC** (10 μM) and commercial lysosome dye (LysoTracker™ Green DND‐26, LTG), the white rectangle is enlarged (scale bar=5 μm). (j) Fluorescence intensity distribution of **MitoSC‐rep** and lysosomes at the position indicated by the white solid line in (i). (k) The Pearson correlation coefficient for **MitoSC‐rep** with lysosomes was 0.86 (*n*=6). **MitoSC‐fun** channel: ex=405 nm, em=420–495 nm; **MitoSC‐rep** channel: ex=561 nm, em=600–640 nm; Mitochondria channel (MTG): ex=488 nm, em=500–550 nm; Lysosomes channel (LTG): ex=488 nm, em=500–550 nm. Quantitative data are expressed as the mean±SEM.

We validated **MitoSC**’s dual localization to the mitochondria and lysosomes upon cellular entry (Figure 2a). First, **MitoSC**’s physicochemical properties were characterized in vitro. Selectivity assays with biologically relevant species, such as Ca^2+^, HSO_3_^−^, GSH, among others, revealed that HS^−^/HSO_3_^−^ significantly weakened **MitoSC**’s fluorescence (Ex: 561 nm) (Figure 2b). Moreover, **MitoSC**′s fluorescence remains unaffected by variations in pH (Figure S18). Subsequently, simulating an HS^−^/HSO_3_^−^‐rich mitochondrial microenvironment in vitro, we measured **MitoSC**’s UV/Vis and fluorescence spectra. The UV/Vis spectra showed a blue shift from 590 nm to 400/425 nm post‐HS^−^/HSO_3_^−^ reaction (Figure S19). From the fluorescence spectra, there was a fluorescence shift with cyanine fluorescence quenching (650 nm) and coumarin fluorescence turn‐on (500 nm) in HS^−^/HSO_3_^−^‐rich environments (Figure 2c, Figure S20). Finally, taking HSO_3_^−^ as an example, titration of **MitoSC** with varying concentrations of HSO_3_^−^ (0–10 μM) revealed a concentration‐dependent and rapid response, with an increase in coumarin fluorescence intensity (405 nm excitation) and a decrease in cyanine fluorescence until near quenching (561 nm excitation) (Figure 2d–e). Linear relationships under both excitation wavelengths corroborated the sensitivity (Figure S21). Thus, **MitoSC** exhibits concentration‐dependent and rapid responsiveness to HSO_3_^−^, disrupting the cyanine's conjugated structure for a rapid fluorescence transition. This high sensitivity ensures **MitoSC**’s quick fluorescence switching in the mitochondria, enabling the visualization of two distinct fluorescent molecules within cells.

To confirm the bicolor fluorescence localization of **MitoSC** in live cells, we assessed its cytotoxicity in HeLa cells. The IC_50_ value was approximately 10 μM (Figure S22); therefore, 10 μM was selected as the working concentration. **MitoSC** entered the cells via an energy‐dependent mechanism (Figure S23), showing blue filamentous/rod‐like structures (ex=405 nm) and red granular ones (ex=561 nm) with partial co‐localization (Figure S24), indicating distinct organelle labeling. Co‐incubation with commercial mitochondrial dye (MitoTracker™ Green FM, MTG^[43]^) confirmed that the blue fluorescence of **MitoSC‐fun** colocalized with MTG‐labeled mitochondria (Figure 2f–h). Similarly, co‐incubation with a lysosomal dye (LysoTracker™ Green DND‐26, LTG^[44]^) confirmed the red fluorescence of **MitoSC‐rep** within lysosomes (Figure 2i–k). To avoid potential interference from the mitophagy process and to further validate the specific localization of **MitoSC** in its corresponding cellular compartments, we employed the mitophagy inhibitor chloroquine (CQ) to prevent the fusion of mitochondria and lysosomes. The results demonstrate that **MitoSC‐fun** and **MitoSC‐rep** exhibited distinct fluorescence distributions in the mitochondria and lysosomes, respectively (Figure S25–26). Furthermore, the localization of **MitoSC** within cells is unaffected by cell type, treatment methods, or other factors (Figure S27). These findings confirm that **MitoSC** possesses excellent dual‐color fluorescence labeling capabilities for mitochondria and lysosomes in living cells, indicating its broad applicability.

In summary, **MitoSC** exists in cells in two forms, **MitoSC‐rep** and **MitoSC‐fun** through specific targeting and functional fluorescence switching, localizing to the lysosomes and mitochondria, respectively, and characterized by red and blue fluorescence, respectively.

### MitoSC Induces Mitophagy by Disrupting the MMP

**Figure 3:**
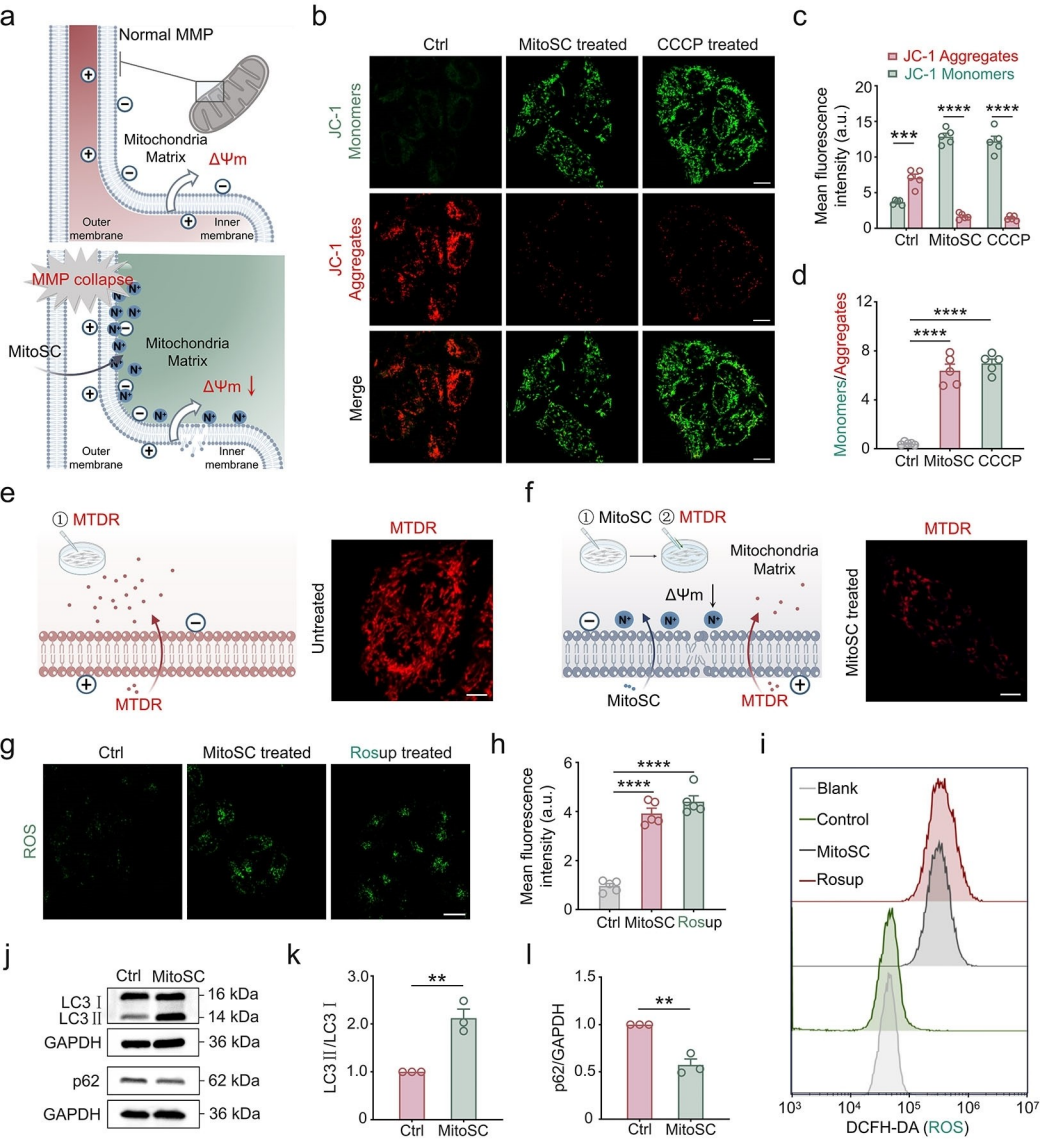
**MitoSC** induces mitophagy by disrupting the mitochondrial membrane potential (MMP). (a) Schematic diagram illustrating the mechanism by which **MitoSC** disrupts the MMP. (b) Detection of MMP after **MitoSC** treatment (10 μM) using JC‐1 (scale bar=10 μm). (c) Mean fluorescence intensity of JC‐1 monomers and JC‐1 aggregates under various treatments (*n*=5). (d) Mean fluorescence intensity ratio of JC‐1 monomers and JC‐1 aggregates under different treatments (*n*=5). (e) Confocal imaging of MTDR‐labeled mitochondria without **MitoSC** treatment (scale bar=5 μm). Created in BioRender. Shao, S. (2024) https://biorender.com/t02t931. (f) Confocal imaging of MTDR‐labeled mitochondria after **MitoSC** (10 μM) treatment (scale bar=5 μm). Created in BioRender. Shao, S. (2024) https://biorender.com/h43h388. (g) Detection of reactive oxygen species (ROS) levels in HeLa cells after **MitoSC** treatment (10 μM) using the ROS probe (DCFH‐DA) (Rosup is a positive control reagent for ROS detection, S0033S‐2, Beyotime, scale bar=10 μm). (h) Mean fluorescence intensity of ROS under different treatments (*n*=5). (i) Flow cytometry detection of ROS levels in HeLa cells following various treatments. (j) western blot analysis of autophagy‐related proteins LC3 and p62 (The corresponding uncropped images can be found in Figure S29). Statistical analysis of the levels of autophagy‐related proteins (k) LC3 and (l) p62 (*n*=3). (JC‐1 monomers channel, ex=488 nm, em=500–550 nm; JC‐1 aggregates channel, ex=561 nm, em=570–600 nm; MTDR channel: ex=640 nm, em=660–700 nm; ROS channel, ex=488 nm, em=500–550 nm) Quantitative data are expressed as the mean±SEM (ns, not significant, *p<0.05, **p<0.01, ***p<0.001, ****p<0.0001).

Next, we delved into the functional activity of **MitoSC** upon its entry into the mitochondria. Mitochondria generate a MMP across their inner membrane owing to the differential distribution of protons, which is crucial for maintaining normal physiological functions and mitochondrial homeostasis.[^45^, ^46^, ^47^] As a positively charged molecule, **MitoSC** responds to the MMP, enters mitochondria, leading to its accumulation and subsequent disruption of the proton gradient, thereby impairing the MMP (Figure 3a). This disruption was evidenced by the disappearance of JC‐1 red aggregates and a significant increase in the number of JC‐1 green monomers^[48]^ in **MitoSC**‐treated cells, resembling the effects of carbonyl cyanide 3‐chlorophenylhydrazone (CCCP), a known MMP disruptor^[49]^ (Figure 3b–d). This indicates that the accumulation of **MitoSC** disrupts the MMP. This finding was further corroborated using an MMP‐dependent probe (MitoTracker™ Deep Red FM, MTDR^[50]^), which showed a marked decrease in mitochondrial labeling following **MitoSC**‐induced MMP disruption (Figure 3e–f, Figure S28).

Subsequent to **MitoSC** treatment, mitochondrial phenotypes similar to those induced by CCCP, including swelling, cristae disruption, loss (Figure S29), and enhanced reactive oxygen species (ROS) production (Figure 3g–h, Figure S30), as confirmed by flow cytometry analysis (Figure 3i). **MitoSC**’s accumulation led to MMP disruption, resulting in mitochondrial damage that triggered mitophagy, as evidenced by increased LC3‐II protein levels and decreased p62 protein levels (Figure 3j–l, Figure S31). These findings confirmed that **MitoSC**‐induced mitochondrial damage prompts cellular clearance via mitophagy. Collectively, **MitoSC** disrupts the mitochondrial integrity by perturbing the MMP upon entry, thereby initiating mitophagy.

### MitoSC Self‐Checks Mitophagy Via Co‐Localization of Dual‐Color Fluorescence in Lysosomes and Mitochondria

**Figure 4:**
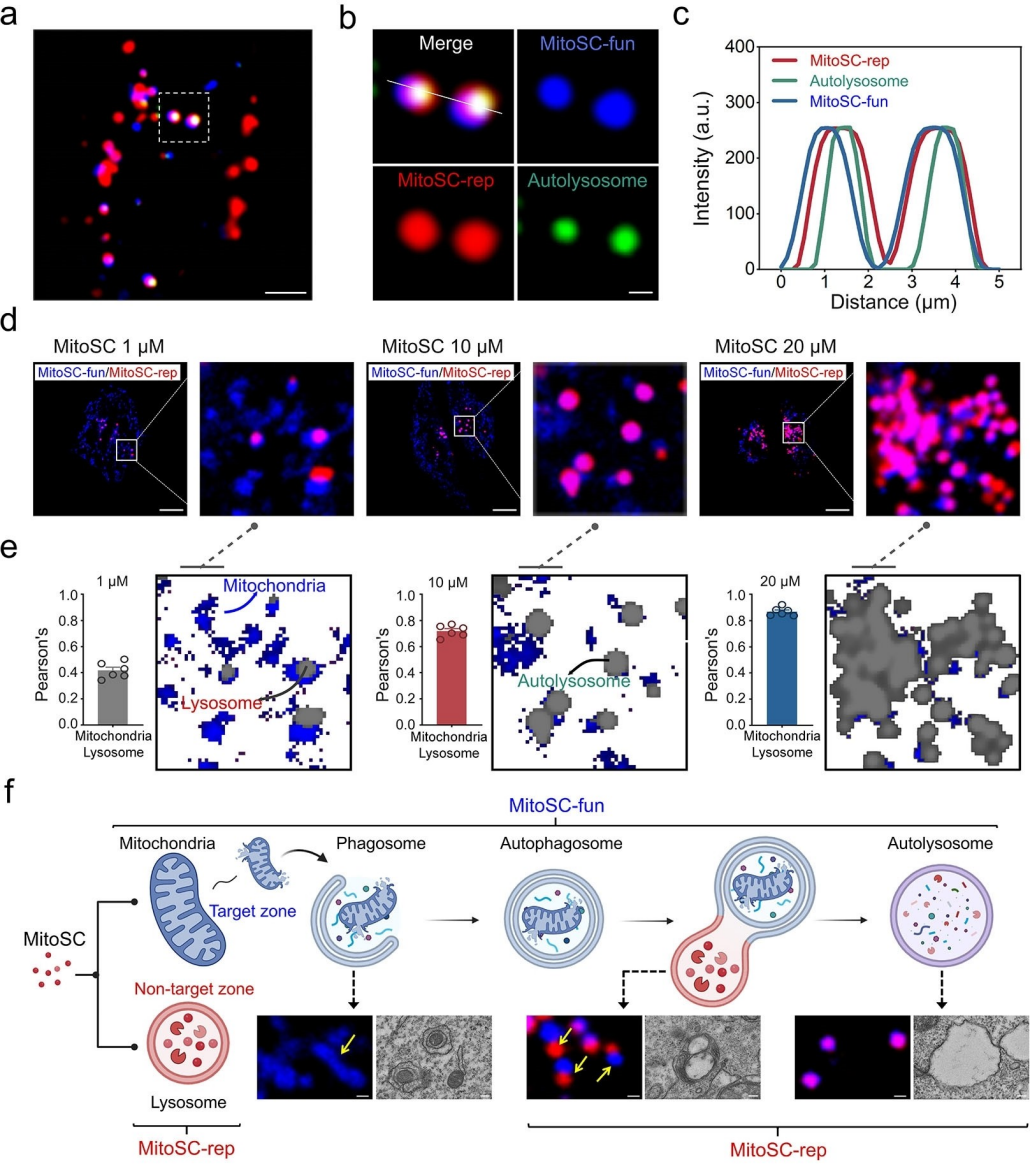
**MitoSC** self‐checks mitophagy via co‐localization of dual‐color fluorescence in lysosomes and mitochondria. (a) Confocal imaging of HeLa cells co‐stained with **MitosSC** (10 μM) and the commercial autolysosome dye (DALGreen‐Autophagy Detection, DALG) (scale bar=5 μm). (b) Enlarged view of the white rectangle in (a) (scale bar=1 μm). (c) Fluorescence intensity distribution of **MitoSC** and DALGreen at the position indicated by the white solid line in (b). (d) Fluorescence images of HeLa cells incubated with different concentrations of **MitoSC** (1, 10 and 20 μM) for 30 min; the white rectangular is enlarged (scale bar=10 μm). (e) The Pearson correlation coefficients for **MitoSC‐fun** with **MitoSC‐rep** at 1, 10 and 20 μM **MitoSC** were 0.42, 0.72 and 0.87 (*n*=6). (f) Schematic diagram illustrating of **MitoSC**’s self‐checking of mitophagy through co‐localization of dual‐color fluorescence in lysosomes (**MitoSC‐rep**) and mitochondria (**MitoSC‐fun**), accompanied by representative fluorescence images (HeLa cells stained with 10 μM **MitoSC** for 30 min, scale bar=0.5 μm) and transmission electron microscope images (HeLa cells treated with 10 μM **MitoSC** for 30 min, scale bar=200 nm) of different stages of mitophagy. Created in BioRender. Shao, S. (2024) https://biorender.com/c64f809 **MitoSC‐fun** channel: ex=405 nm, em=420–495 nm; **MitoSC‐rep** channel: ex=561 nm, em=600–640 nm; Autolysosome channel (DALG): ex=488 nm, em=500–550 nm. Quantitative data are expressed as the mean±SEM.

Upon examining the blue fluorescence (**MitoSC‐fun**) localized in the mitochondria (Figure 2f–h) and the red fluorescence (**MitoSC‐rep**) in lysosomal regions (Figure 2i–k), with partial co‐localization observed between these dual colors (Figure S24), our findings confirmed that **MitoSC** not only induces mitophagy within the mitochondrial targets but also tracks mitophagy via lysosomal reporting. This was further validated by the high coincidence of **MitoSC‐fun and MitoSC‐rep** fluorescence with the commercial autolysosome dye, DALGreen‐Autophagy Detection, DALG^[51]^ (Figure 4a–c). Furthermore, when compared to mature mitophagy inducers such as CCCP and carbonyl cyanide 4‐(trifluoromethoxy)phenylhydrazone (FCCP) under identical administration conditions, **MitoSC** demonstrates a more pronounced effect on mitophagy induction (Figure S32).

Additionally, we investigated the effect of varying **MitoSC** concentrations on self‐checking mitophagy. At 1 μM **MitoSC**, there was minimal overlap between the lysosomes (red) and mitochondria (blue), indicating low mitophagy levels. Increasing the concentration to 10 and 20 μM, resulted in enhanced mitophagy, as evidenced by increased co‐localization of red (**MitoSC‐rep**) and blue (**MitoSC‐fun**) fluorescence (Figure 4d–e). This concentration‐dependent enhancement of mitophagy is consistent with **MitoSC**’s impact on HeLa cell viability (Figure S22). Furthermore, extended incubation with **MitoSC** intensified the observed mitophagy (Figure S33), suggesting that mitophagy levels are modulated by both **MitoSC** concentration and incubation time, with **MitoSC‐rep** and **MitoSC‐fun** reflecting varying degrees of mitophagy damage. Fluorescence microscopy and transmission electron microscopic images also illustrated the different stages of mitophagy following **MitoSC** treatment (Figure 4f).

Overall, these results demonstrate that **MitoSC**, while inducing mitophagy, also provides a self‐reporting mechanism for mitophagy levels through the overlap of blue (**MitoSC‐fun**) and red (**MitoSC‐rep**) fluorescence. This feature makes **MitoSC** a powerful tool for visualizing and monitoring therapeutic progression in mitophagy research.

### MitoSC Exhibits a Strong Anti‐Tumor Ability on 3D HeLa Spheroids and a Mouse Xenograft Model

**Figure 5:**
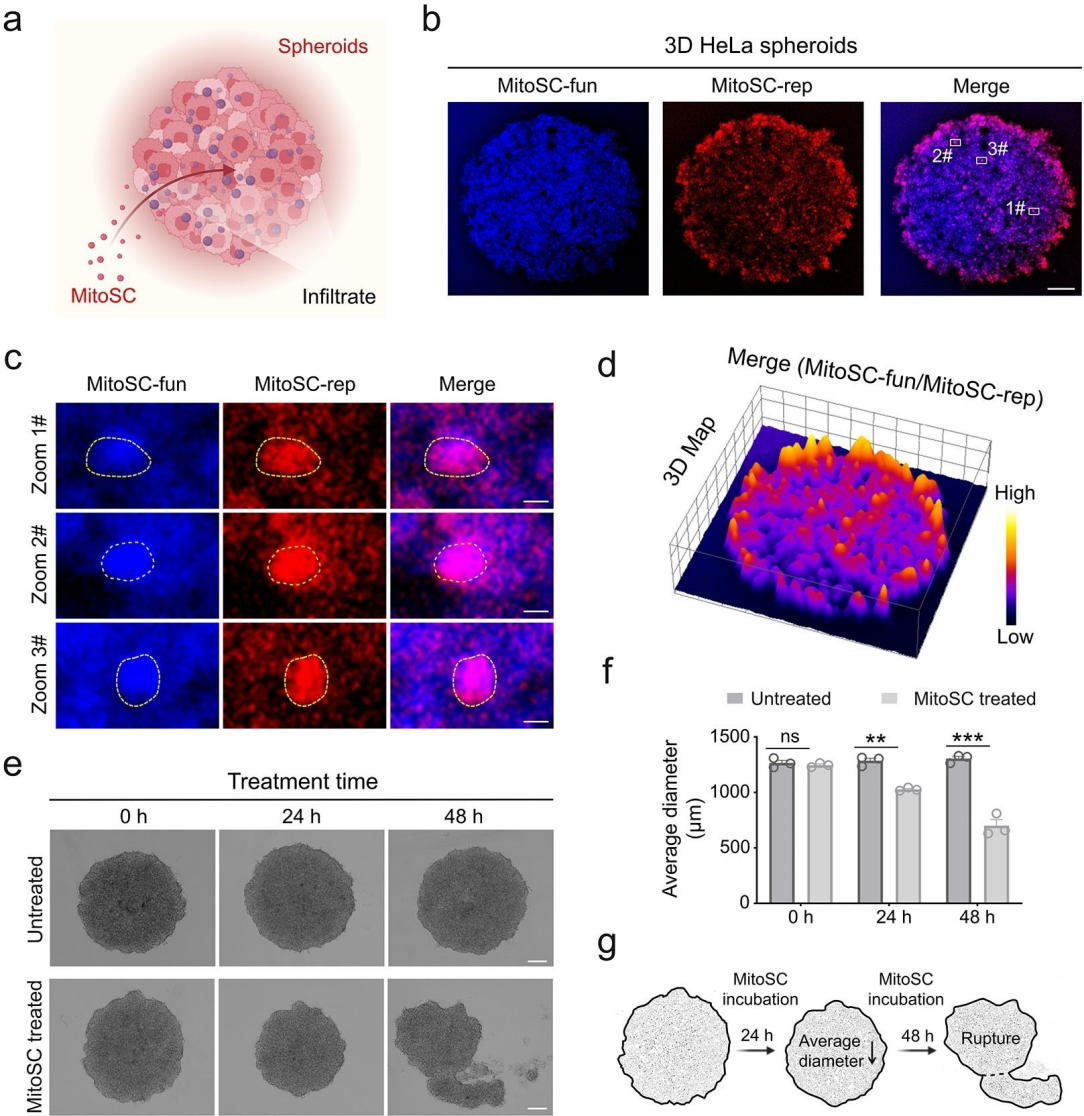
**MitoSC** infiltrates 3D HeLa spheroid and inhibits its growth. (a) Schematic diagram illustrating **MitoSC** infiltration of 3D HeLa spheroid. Created in BioRender. Shao, S. (2024) https://biorender.com/b52d873. (b) Representative fluorescence images of 3D HeLa spheroids incubated with **MitoSC** (10 μM) for 30 min (scale bar=200 μm). (c) Enlarged view of the white rectangle in (b) (Dotted circles indicate the accumulation of **MitoSC**) (scale bar=10 μm). (d) 3D map of the merged image in (b) showing the distribution of **MitoSC** within 3D HeLa spheroids. (e) Representative images of 3D HeLa spheroids at 0, 24, and 48 h with or without **MitoSC** treatment (scale bar=200 μm). (f) Quantitative analysis of the average diameter of 3D HeLa spheroids at 0, 24 and 48 h with or without **MitoSC** treatment (*n*=3). (g) Schematic diagram depicting the state of 3D HeLa spheroids incubation with **MitoSC** for 48 h. Created in BioRender. Shao, S. (2024) https://biorender.com/w51t396 Quantitative data are expressed as the mean±SEM (ns, not significant, *p<0.05, **p<0.01, ***p<0.001, ****p<0.0001).

Given the complex 3D architecture of tumor tissues, which includes numerous cancer cells,[^52^, ^53^, ^54^] we developed 3D HeLa spheroids (tumoroids) to evaluate **MitoSC**’s efficacy in inhibiting tumor proliferation. Notably, **MitoSC** effectively infiltrated the spheroid (Figure 5a–d), and potently suppressed spheroid growth (Figure 5e–g), highlighting its potent tumor‐suppressing capability in vitro. Moreover, compared to MCF‐10 A cells (breast epithelial cells), **MitoSC** demonstrates more significant inhibitory effects on the proliferation of MCF‐7 cells (breast cancer cells). This indicates that **MitoSC** has a more potent targeted therapeutic effect on tumor cells with high MMP (Figure S34).

**Figure 6:**
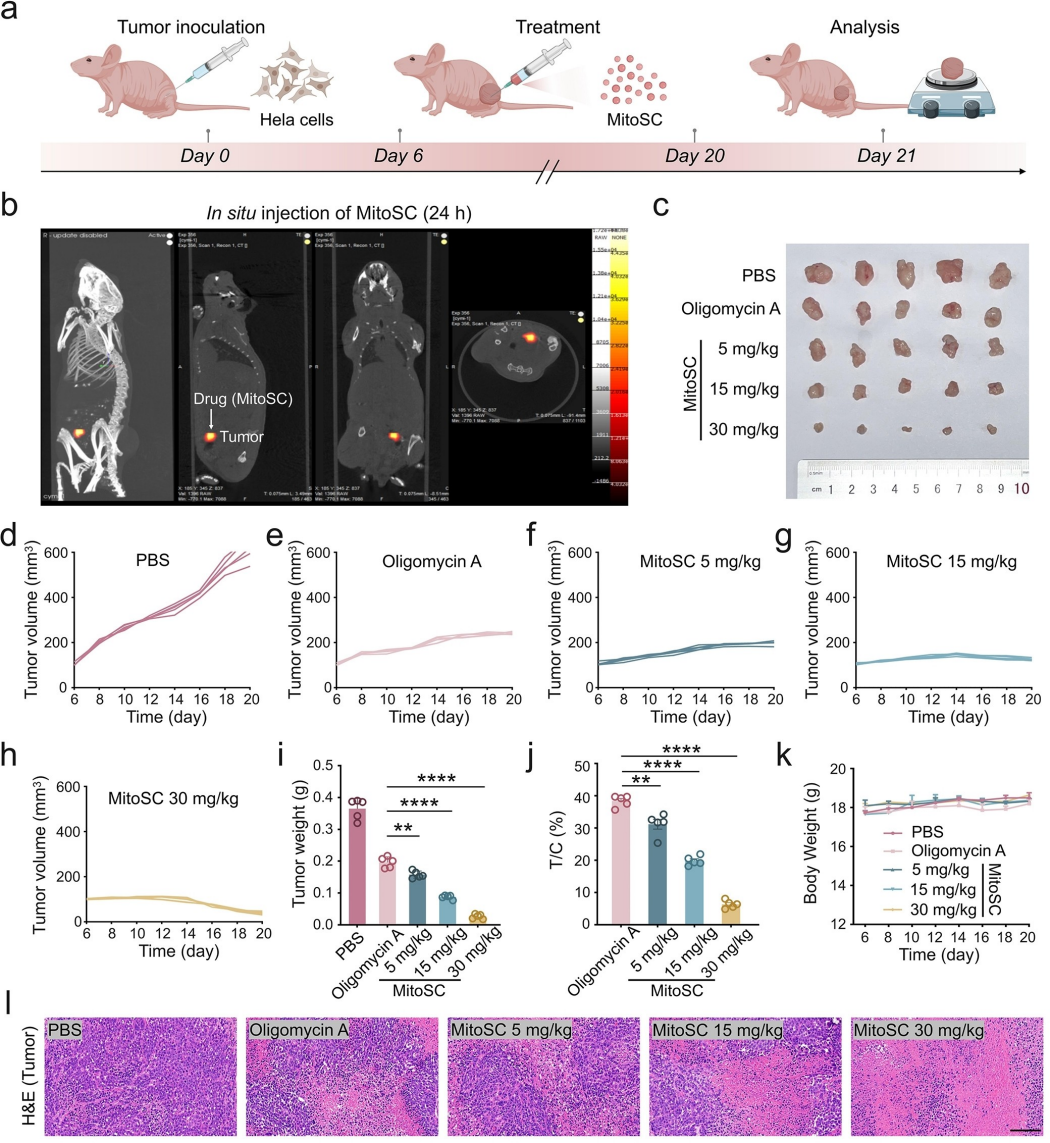
**MitoSC** exhibits a strong antitumor activity in a mouse xenograft model. (a) Timeline of animal experiment. Created in BioRender. Shao, S. (2024) https://biorender.com/b47d866. (b) 3D fluorescence tomography imaging (InSyTe FLECT/CT) of small animal after in situ injection of **MitoSC** (10 μM, 100 μL) (ex=561 nm, em=570–640 nm). (c) Digital photographs of tumors excised from five mouse groups after different treatments. (d–h) Changes in tumor volume during treatment in the following groups: (d) PBS, (e) Oligomycin A, (f) **MitoSC** (5 mg/kg), (g) **MitoSC** (15 mg/kg), and (h) **MitoSC** (30 mg/kg) (*n*=5). (i) Data analysis of tumor weights dissected from five mouse groups (*n*=5). (j) Relative tumor proliferation rate in four mouse groups on day 20 (T/C [%]≤40 % and statistically processed *P* <0.05 were valid) (*n*=5). (k) Changes in body weight during treatment in the five mouse groups (*n*=5). (l) Representative H&E staining images of tumor after different treatments (scale bar=100 μm). Quantitative data are expressed as the mean±SEM (ns, not significant, *p<0.05, **p<0.01, ***p<0.001, ****p<0.0001).

Furthermore, we evaluated the in vivo efficacy of **MitoSC** for antitumor activity and selected Oligomycin A as the positive control. Oligomycin A inhibits oxidative phosphorylation, resulting in a reduction in MMP (Figure 6a). Our initial investigation demonstrated significant tumor accumulation of **MitoSC** (Figure 6b). Compared to the positive control, Oligomycin A,[^55^, ^56^] **MitoSC** showed superior therapeutic outcomes (Figure 6c, Figure S35), as evidenced by substantial reductions in tumor volume (Figure 6d–h, Figure S36), tumor weight (Figure 6i), and proliferation (Figure 6j, Figure S37), without affecting mouse body weight (Figure 6k). Histological analysis following **MitoSC** treatment revealed extensive necrosis, which increased with higher treatment concentration (Figure 6l), while other vital organs (heart, liver, spleen, lungs, and kidneys) remained unaffected (Figure S38). In summary, these results establish **MitoSC**’s potent antitumor capabilities.

## Conclusions

In this study, we introduced a novel “one‐two punch” drug design strategy targeting the MMP and successfully developed **MitoSC**, a dual‐color and dual‐targeting agent that both induces and self‐checks mitophagy. Functional validation studies have confirmed that, unlike existing mitophagy drugs, **MitoSC** is distinguished by its innovative design, which integrates both the induction and self‐checking of mitophagy. Within cells, **MitoSC** transforms into **MitoSC‐fun** in the targeted zone and into **MitoSC‐rep** for non‐target zone biological reporting, eliminating the need for fluorescent tags commonly used in conventional validation methods. This approach circumvents interference from exogenous probes and streamlines the verification process. Moreover, we investigated **MitoSC**’s antitumor properties, demonstrating its accumulation in mitochondria to trigger organelle‐mediated cell death signals and enhance precise subcellular targeting of tumor cells. In vivo experiments further confirmed **MitoSC**’s potent tumor‐suppressive effects, underscoring its potential as a cancer therapeutic. Importantly, the “one‐two punch” strategy offers new perspectives and methodologies in drug discovery. Traditional drug evaluation is complicated by the presence of exogenous substances, leading to inaccurate efficacy assessments. This strategy surpasses the limitations of conventional drug development by extending optimization beyond initial functionality to include subsequent validation phases. While our research successfully segregates target‐zone functionality from biological reporting in theranostic drugs, current limitations in subcellular drug quantification technologies hinder precise determination of their respective doses. Advancements in super‐resolution quantitative chemical imaging technologies are necessary for future clarification.

In summary, **MitoSC** exemplifies a paradigm‐shifting drug design that concurrently exerts function and reports biological activity. By integrating upstream target‐specific fluorescence with downstream non‐target reporter fluorescence, **MitoSC** not only activates but also self‐checks mitophagy, visually representing the dynamic changedds in key organelles (mitochondria and lysosomes) involved in the process. This provides invaluable insights into the subcellular dynamics of mitophagy drugs. The “one‐two punch” strategy opens new avenues in drug design, poised to propel the field forward and enrich clinical pipelines with innovative therapeutic options.

## Conflict of Interests

The authors declare no conflict of interest.

1

## Supporting information

As a service to our authors and readers, this journal provides supporting information supplied by the authors. Such materials are peer reviewed and may be re‐organized for online delivery, but are not copy‐edited or typeset. Technical support issues arising from supporting information (other than missing files) should be addressed to the authors.

Supporting Information

## Data Availability

The data that support the findings of this study are available in the supplementary material of this article.
