# Supplementary material for: A Small‐Molecule Drug for the Self‐Checking of Mitophagy
Source: Angew Chem Int Ed Engl. 2025 Jan 22;64(10):e202421269. doi: 10.1002/anie.202421269 (PMC11894447; doi:10.1002/anie.202421269)
Supplement: Supplementary file 1 — Supporting Information [file ANIE-64-e202421269-s001.pdf]

Angewandte Chemie
Eine Zeitschrift der Gesellschaft Deutscher Chemiker

Supporting Information

A Small-Molecule Drug for the Self-Checking of Mitophagy

Y. Gao, Q. Bai, Y. Ren, X. Shao, M. Zhang, L. Wu, S. E. Lewis, T. D. James, X. Chen*, Q. Chen**

Supporting Information

Yanan Gao[a]+, Qingjie Bai[a]+, Youxiao Ren[a]+, Xintian Shao[a], Mengrui Zhang[a], Luling Wu[c]*, Simon E. Lewis[c], Tony D. James[c, d], Xiaoyuan Chen[b, e, f, g, h, i]*, Qixin Chen[a, b, f, g]*

[a] Ms. Y. Gao, Mr. Q. Bai, Ms. Y. Ren, Dr. X. Shao, Mr. M. Zhang, Prof. Q. Chen
State Key Laboratory of Advanced Drug Delivery and Release Systems, School of Pharmaceutical Sciences, Neck-Shoulder and Lumbocrural Pain Hospital, Medical Science and Technology Innovation Center, Shandong First Medical University & Shandong Academy of Medical Sciences, Jinan, Shandong 250117, PR China.

[b] Prof. X. Chen, Prof. Q. Chen
Departments of Diagnostic Radiology, Chemical and Biomolecular Engineering, and Biomedical Engineering, Yong Loo Lin School of Medicine and College of Design and Engineering, National University of Singapore, Singapore, 119074, Singapore.
E-mail: chen.shawn@nus.edu.sg (X. Chen); dnrv31@nus.edu.sg (Q. Chen).

[c] Dr. L. Wu, Prof. Simon E. Lewis, Prof. T. D. James
Department of Chemistry
University of Bath
Bath BA2 7AY, U.K.
E-mail: lw960@bath.ac.uk.

[d] Prof. T. D. James
School of Chemistry and Chemical Engineering, Henan Normal University, Xinxiang 453007, People's Republic of China.

[e] Prof. X. Chen
Clinical Imaging Research Centre, Centre for Translational Medicine, Yong Loo Lin School of Medicine, National University of Singapore, Singapore 117599, Singapore.

[f] Prof. X. Chen, Prof. Q. Chen
Nanomedicine Translational Research Program, Yong Loo Lin School of Medicine, National University of Singapore, Singapore 117597, Singapore.

[g] Prof. X. Chen, Prof. Q. Chen
Theranostics Center of Excellence (TCE), Yong Loo Lin School of Medicine, National University of Singapore, 11 Biopolis Way, Helios, Singapore 138667.

[h] Prof. X. Chen
Institute of Molecular and Cell Biology, Agency for Science, Technology, and Research (A*STAR), 61 Biopolis Drive, Proteos, Singapore, 138673, Singapore.

[i] Prof. X. Chen
Department of Pharmacy and Pharmaceutical Sciences, National University of Singapore, Lower Kent Ridge Road, 4 Science Drive 2, 117544, Singapore

+ These authors contributed equally to this work.

Materials and methods

General materials

Reagents and consumables for synthesis, including 7-(diethylamino)-2-oxo-2H-chromene-3-carbaldehyde and 4-(2-aminoethyl) morpholine, were purchased from Macklin and Aladdin in Shanghai, China. 5-carboxy-1-ethyl-2,3,3-trimethyl-3H-indol-1-ium iodide (Mito 1) was purchased from Dalian Monty Technology Co., Ltd. Fetal bovine serum (FBS) was sourced from VivaCell Shanghai. Dulbecco's modified Eagle's medium (DMEM), Penicillin-streptomycin (10,000 units/ml), Trypsin-EDTA phenol-free medium, and other cell culture reagents were acquired from Gibco BRL in Grand Island, NY, USA. LTR (LysoTracker™ Green DND-26, LTG), MTG (MitoTracker™ Green FM, MTG) and MTDR (MitoTracker™ Deep Red FM, MTDR) were sourced from Invitrogen in Eugene, Oregon, USA. DALG (DALGreen-autophagy detection) was sourced in Dojindo Laboratories, Japan. HeLa cells were generously provided by Fengshan Wang's lab at Shandong University.

Synthesis and characterization of MitoSC

7-(Diethylamino)-2-oxo-2H-chromene-3-carbaldehyde (0.5 g, 2.0 mmol, 1 eq.) and 5-carboxy-1-ethyl-2,3,3-trimethyl-3H-indol-1-ium iodide (Mito 1) (1.0 g, 2.8 mmol, 1.4 eq) were dissolved in ethanol (25 mL) under a nitrogen protection atmosphere. Piperidine (60 μL, 0.05 g, 0.3 eq.) was then added, the reaction mixture was stirred at 80°C for 16 h. The reaction mixture was cooled down to room temperature, followed by purification using silica gel chromatography with the eluent (MeOH/DCM = 1/20) to yield a dark khaki solid (1.0 g, 1.7 mmol yield 85%). 50 mg of the solid was taken in a bottle, 5 mL of pure water was added, and the mixture was sonicated for 5 min, followed by filtration of the solid. This process was repeated three times to obtain pure compound Mito 2 (5 mg, 0.085 mmol yield 4.3%).

Compound Mito 2 (0.2 g, 0.341 mmol, 1 eq.), chloro-*N*,*N*,*N*',*N*'-tetramethylformamidinium hexafluorophosphate (TCFH) (0.143 g, 0.511 mmol, 1.5 eq) and DIEA (0.132 g, 1.02 mmol, 3.0 eq) were dissolved in ACN (1 mL) under dark conditions. 4-(2-aminoethyl) morpholine (6.43 μL, 0.409 mmol, 1.2 eq.) was then added, the reaction mixture was stirred at room temperature for 2.5 h. The reaction mixture was concentrated under reduced pressure, frozen at -20°C for 12 h, and washed three times with MeOH/MTBE = (1/10). The resulting oil was dissolved in 2 ml MeOH and frozen at -20°C for 12 h. The solids are filtered out and dried to obtain the target product **MitoSC** (15 mg, 0.0214 mmol, yield 6.3%).

Cell culture

HeLa cells were cultured in Dulbecco's modified Eagle's medium (#11965118, DMEM, Thermo Fisher Scientific) supplemented with 10% fetal bovine serum (#26140079, FBS, Thermo Fisher Scientific), penicillin (100 units/ml), and streptomycin (100 μg/ml; #15140163, 10,000 units/ml, Thermo Fisher Scientific) in a 5% CO_2 humidified incubator at 37°C.

The cytotoxicity assay

HeLa cells were seeded in 96-well plates at a density of 7 × 10^3 cells/well in DMEM with 10% FBS and incubated at 37°C in 5% CO_2 for 24 h. The medium was then replaced with 100 μL fresh medium containing different concentrations of **MitoSC** (0.5, 1.0, 2.5, 5.0, 10.0, 20.0 and 30.0 μM). Following incubation for 1, 3, 12 or 24 h, 10 μL of CCK8 solution was added to each well, and the plates were incubated for an additional 2 h under the same conditions. The absorbance of each well at 450 nm was determined by enzyme-linked immunosorbent assay plate reader. Cell viability (%) = [(As - Ab)/(Ac - Ab)] × 100%, where As represents the absorbance of the experimental group, Ac represents the absorbance of the control group, and Ab represents the absorbance of the blank group.

Handling and staining of live cells

A total of 2 × 10^5 cells were seeded onto a glass bottom culture dish with a diameter of 3.5 cm and incubated with 2 mL of DMEM supplemented with 10% FBS for 24 h. The cells were then stained with 10 μM **MitoSC** for 30 min, followed by staining with 100 nM MitoTracker™ Green FM (#M7514, MTG, Invitrogen), 200 nM LysoTracker™ Green DND-26 (#L7526, LTG, Invitrogen) or 200 nM DALGreen-Autophagy Detection (#D657, DALG, Dojindo) at 37°C for another 30 min, Then, the cells were washed three times with pre-warmed PBS and twice with fresh DMEM. Finally, cells were cultured in phenol-red-free medium (#1894117, Gibco) and observed using a confocal laser scanning microscopy (LSM-980, Carl Zeiss, Inc.).

Confocal laser scanning microscopy imaging

The images were acquired using an LSM-980 confocal laser scanning microscope (Carl Zeiss, Inc.) equipped with a 63×/1.49 numerical aperture oil immersion objective lens. Data analysis was performed using ZEN software (version 3.5.093, Carl Zeiss, Inc.) and ImageJ software (version 1.51j8, National Institutes of Health).

Cellular uptake assay

HeLa cells were incubated with 10 μM **MitoSC** under different conditions. 37°C: The cells were incubated with **MitoSC** at 37°C for 30 min. 4°C: The cells were incubated with **MitoSC** at 4°C for 30 min. MI (Metabolic inhibitors): The cells were pre-incubated with 50 mM 2-deoxy-D-glucose and 5 μM oligomycin in FBS-free DMEM at 37°C for 1 h and followed by incubation with **MitoSC** at 37°C for 30 min. NH_4Cl: The cells were pre-incubated with 50 mM NH_4Cl in FBS-free DMEM at 37°C for 1 h, followed by incubation with **MitoSC** at 37°C for 30 min. After these treatments, the cells were observed using

a confocal laser scanning microscope (LSM-980, Carl Zeiss, Inc.).

Western Blot

After processing, the cell samples were washed twice with precooled PBS, centrifuged at 4°C The supernatant was discarded, and the cells were then then lysed on ice with RIPA lysis buffer containing protease and phosphatase inhibitors for 30 min. During lysis, ultrasonic disruption was performed. The lysates were centrifuged at 12,000 rpm for 15 min at 4°C, and the supernatant was collected the protein sample. A portion of the supernatant was used to determine the total protein concentration using a BCA protein assay kit. Subsequently, the following steps were performed: gel loading and electrophoresis, transfer of proteins from the gel to a membrane, blocking, antibody incubation, and detection. For analysis, the LC3 ratio (LC3-II/LC3-I) and p62 levels (p62/GAPDH) were assessed. Data analysis was conducted based on the results from three independent experiments.

Evaluation of the permeability of MitoSC in 3D HeLa spheroids

HeLa cells seeded at a density of $1{\times}10^4$ per well into a U-shaped 96-well plate (#4515, Thermo Fisher Scientific), and incubated until tumor spheroids were formed. The spheroids were then incubated with 10 μM **MitoSC** for 30 min, Following incubation, the medium was replaced with phenol red-free culture medium. Images of the tumor spheroids were captured using an inverted microscope (IX73, Olympus) with a 20x objective lens.

Evaluation of MitoSC inhibiting 3D HeLa spheroids growth

HeLa cells were seeded at a density of $1{\times}10^4$ per well in a U-shaped 96-well plate (#4515, Thermo Fisher Scientific), and incubated until tumor spheroids formed. The spheroids were then treated with 10 μM **MitoSC**, and images were captured at 0, 24 and 48 h post-treatment using an inverted microscope (IX73, Olympus), with a 20× objective lens.

***In vivo* evaluation of the antitumor efficacy of MitoSC**

Female BALB/c nude mice were provided by Beijing Weitong Lihua Experimental Animal Technology Co., Ltd. with animal license SCXK (Beijing) 2021-0006. The animal experiments were approved by the Ethics Committee of Experimental Animals at Shandong First Medical University (2023S 6028). To induce tumor formation, a 200 μL suspension of HeLa cells (5×10^6 cells per mouse) was subcutaneously injected into the right hind limb of female BALB/c nude mice. When the tumors reached a volume of approximately 100 mm^3, the mice were randomly allocated into five groups (n = 5 per group). The groups received in situ injections of different treatments: the control group was administered PBS, one group received Oligomycin A (5 mg/kg), and the remaining three groups were treated with varying doses of **MitoSC** (5 mg/kg, 15 mg/kg, or 30 mg/kg). Injections were administered every two days, with tumor volume and body weight recorded prior to each injection. At conclusion of the study, the mice were euthanized, tumors were excised, and their weights were recorded. The tumor volume was determined using the following formula (1):

$$\text{Volume} = \frac{\text{Length} \times \text{Width}^2}{2} \quad (1)$$

The relative tumor proliferation rate T/C (%) was determined using the following formula (2):.

$$T/C\ (\%) = T_{RTV}/C_{RTV} \times 100\% \quad (2)$$

where T_{RTV} is the relative tumor volume in the treatment group, and C_{RTV} is the relative tumor volume in the control group. A T/C (%) > 40% was considered invalid, while T/C (%) ≤ 40% and statistically processed $P < 0.05$ was valid.
Relative tumor volume (RTV) was calculated as:

$$RTV = V_t/V_0$$

where V_0 is the tumor volume measured at the time of at the start of treatment, and V_t is the tumor volume at each measurement.

3D fluorescence tomography imaging of small animal

Mice were injected with **MitoSC** *in situ* (10 μM, 100 μL) and normally fed for 24 h. Prior to the experiment, the mice were placed in an anesthesia chamber with an isoflurane gas concentration of 2% and a flow rate of 1.5 LPM to rapidly induce

anesthesia. One minute later, the mice were confirmed to be in a state of deep anesthesia. Subsequently, the mice were placed in a 30 mm diameter Image Cell and then onto the animal bed of the equipment. Fluorescence imaging (ex = 561 nm) and CT scanning were performed under preset parameters. Continuous anesthesia was maintained throughout the experiment.

Histological evaluation of tumors and major organs

Tumors and organ samples were collected with neither the thickness not exceeding 2 mm and a size not exceeding 5 × 5 mm. Samples were immediately fixed in fixative solution, followed by dehydration, clearing, paraffin embedding, and sectioning to produce paraffin sections. The sections were dewaxed and stained with hematoxylin and eosin (H&E). Digital imaging of the sections was performed using a slide scanner (PANNORAMIC SCAN II, Hungary).

Data analysis

Statistical analysis was performed using Origin 2021, GraphPad Prism 8 and ImageJ V.1.51j8. Data were tested for normal distribution using a normality test and presented as mean ± SEM. For data following a normal distribution, statistical comparison were performed using a t-test. For non-normally distributed data, comparisons were conducted using the Mann-Whitney test. The significance levels were donated as ns (not significant), *$P < 0.05$, **$P < 0.01$, ***$P < 0.001$, and ****$P < 0.0001$. Sample sizes and significance levels are indicated in the figure legends.

Supplementary Figures

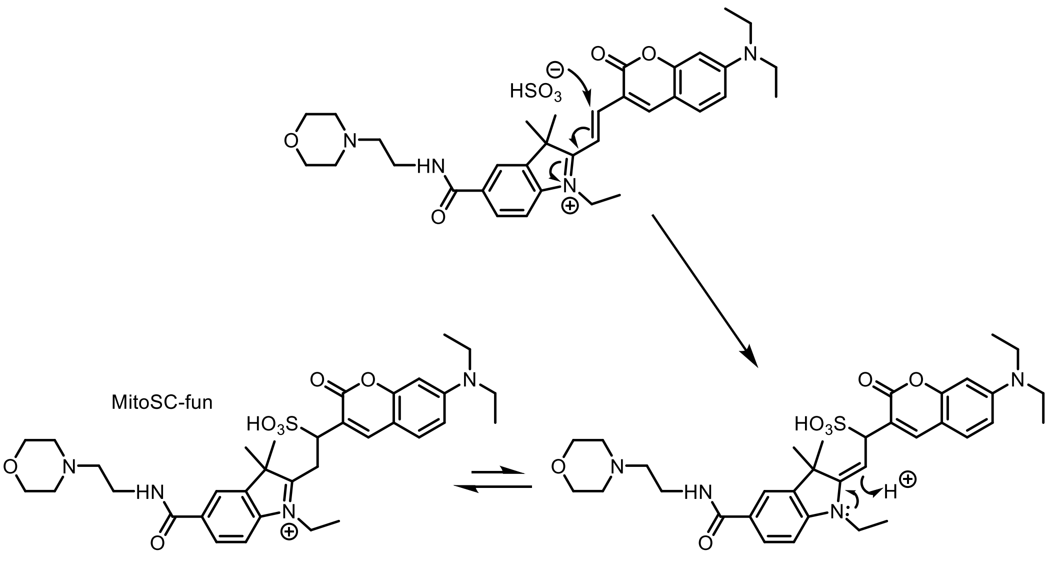

Figure S1. The proposed reaction mechanism between **MitoSC** and HSO_3^-.

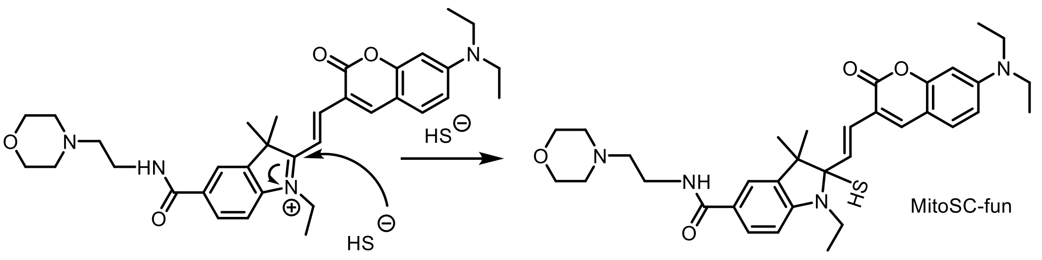

Figure S2. The proposed reaction mechanism between **MitoSC** and HS^-.

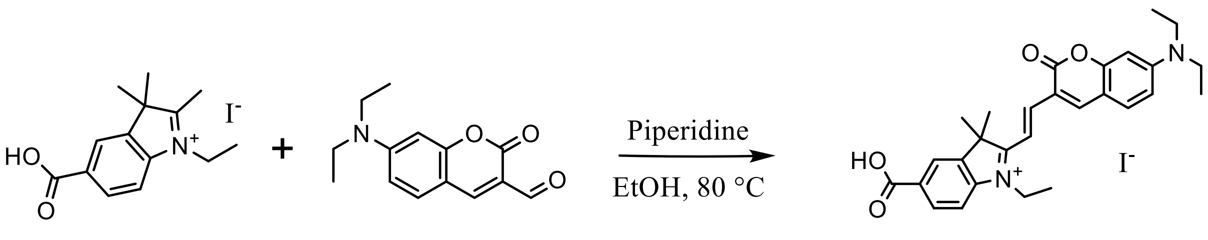

Figure S3. Synthesis of Mito 2.

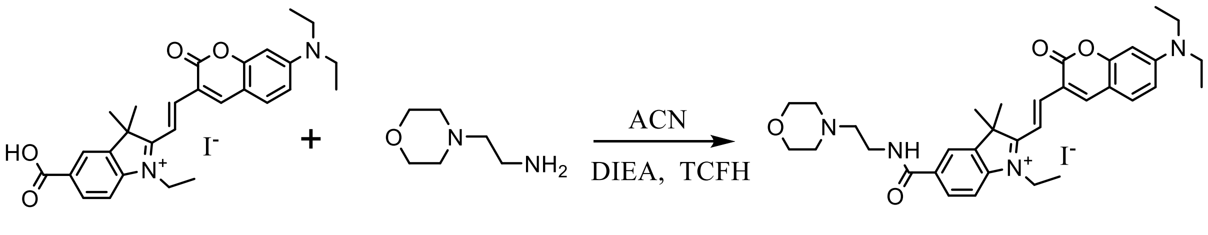

Figure S4. Synthesis of **MitoSC**.

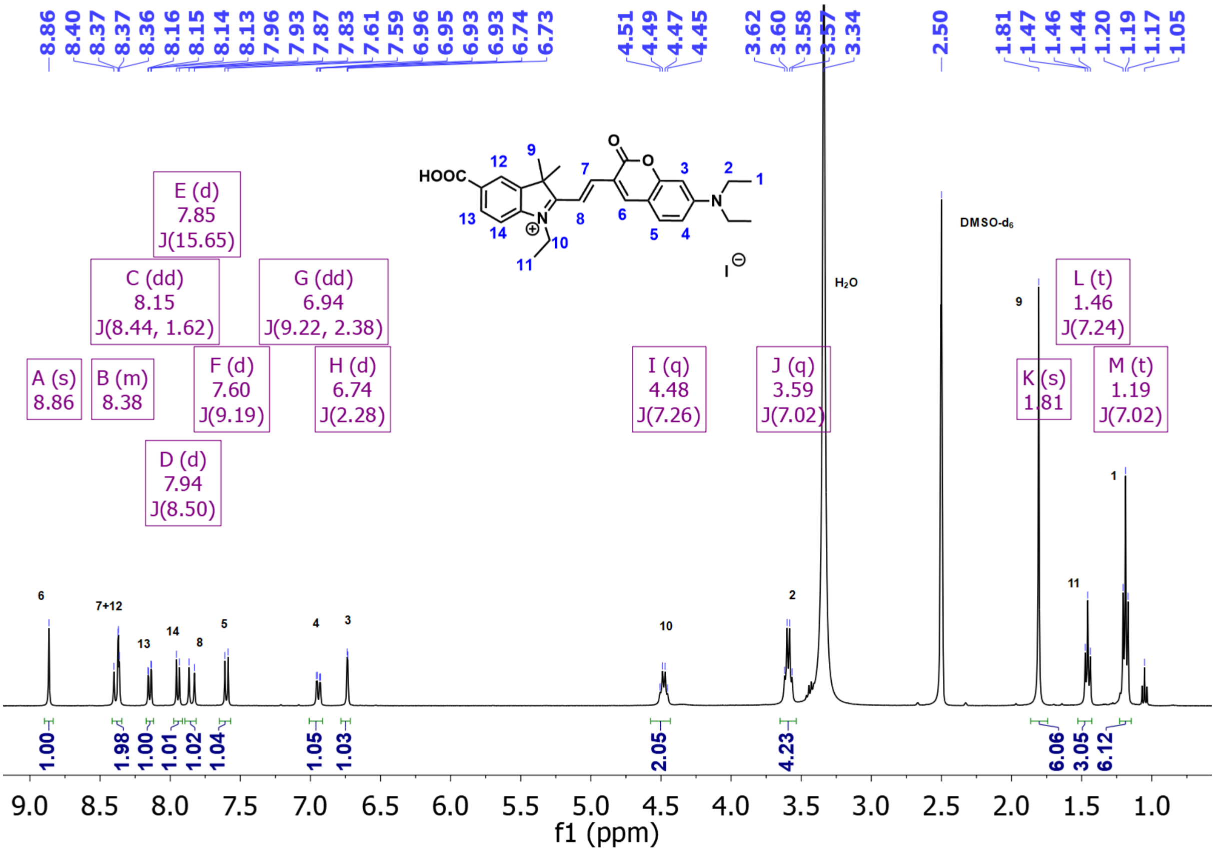

Figure S5. ^{1}H-NMR spectrum of Mito 2 in DMSO-d_6.^{1}H NMR (400 MHz, DMSO-d_6) δ 8.86 (s, 1H, H6), 8.40-8.36 (m, 2H, H7 and H12), 8.15 (dd, *J* = 8.4, 1.6 Hz, 1H, H13), 7.94 (d, *J* = 8.5 Hz, 1H, H14), 7.85 (d, *J* = 15.6 Hz, 1H, H8), 7.60 (d, *J* = 9.2 Hz, 1H, H5), 6.94 (dd, *J* = 9.2, 2.4 Hz, 1H, H4), 6.74 (d, *J* = 2.3 Hz, 1H, H3), 4.48 (q, *J* = 7.3 Hz, 2H, H10), 3.59 (q, *J* = 7.0 Hz, 4H, H2), 1.81(s, 6H, H9), 1.46 (t, *J* = 7.2 Hz, 3H, H11), 1.19 (t, *J* = 7.0 Hz, 6H, H1).

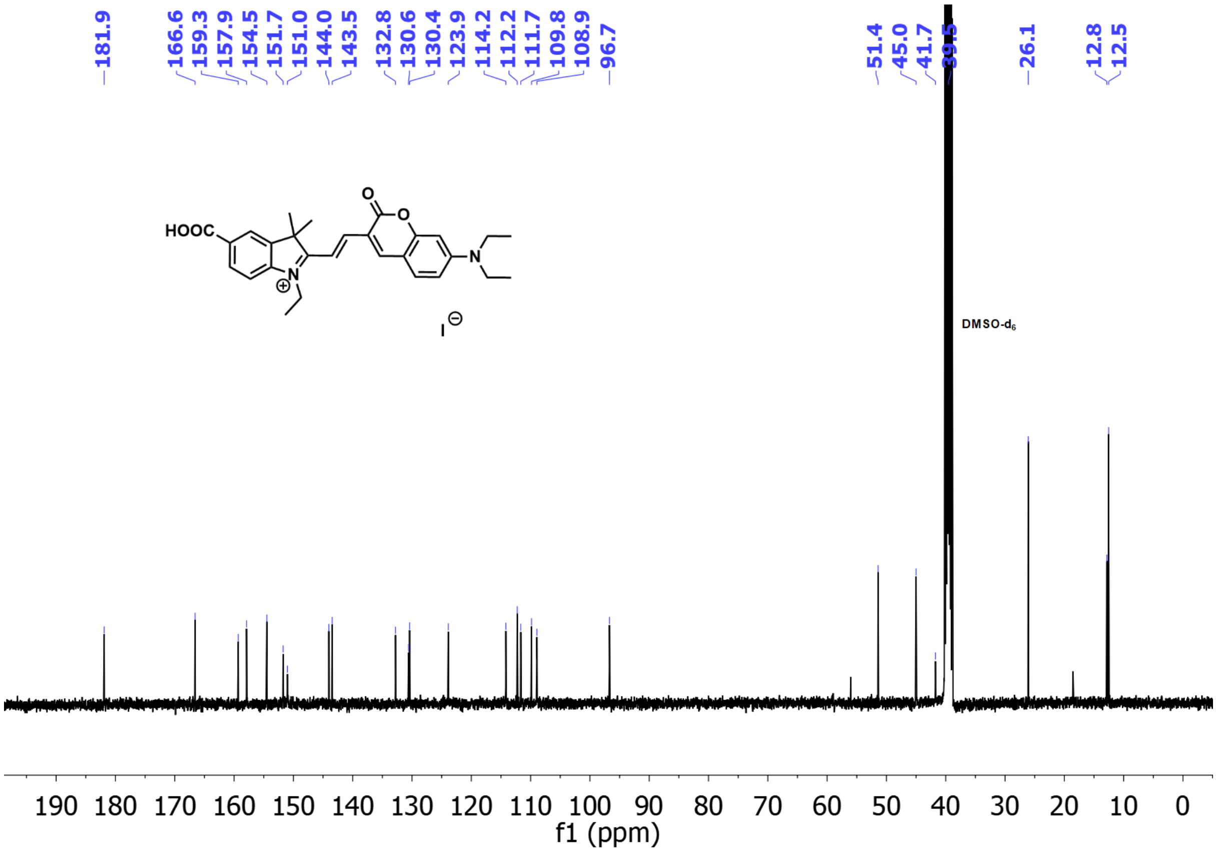

Figure S6. ^{13}C-NMR spectrum of Mito 2 in DMSO-d_6.

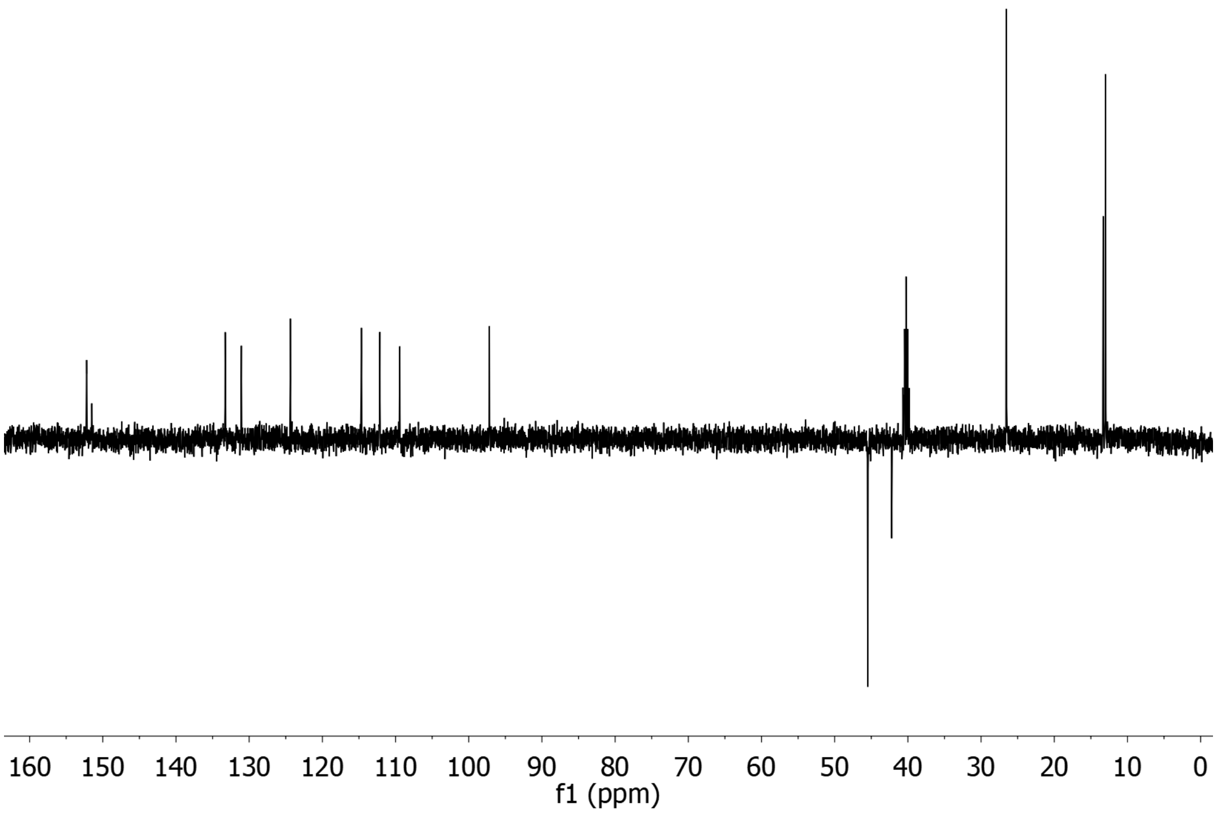

Figure S7. DEPT135 spectrum of Mito 2 in DMSO-d_6.

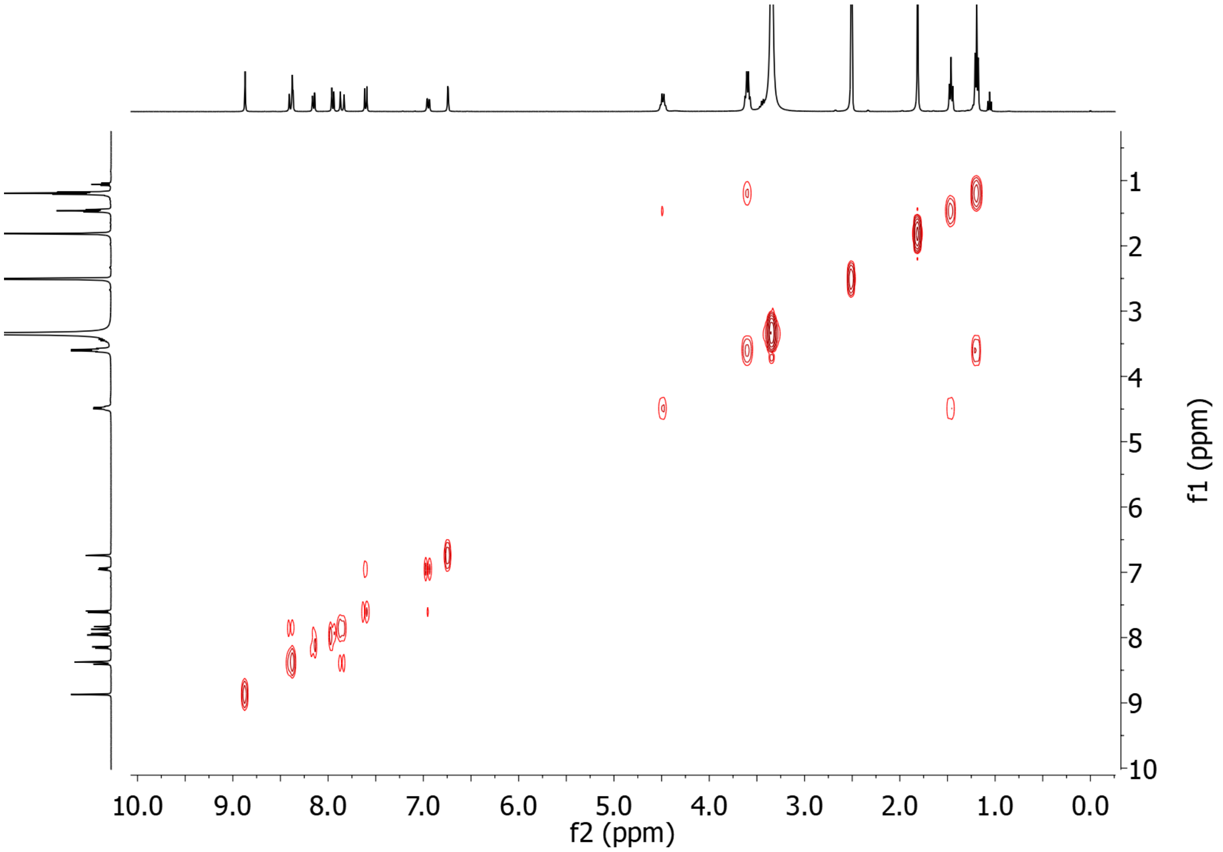

Figure S8. COSY spectrum of Mito 2 in DMSO-d_6.

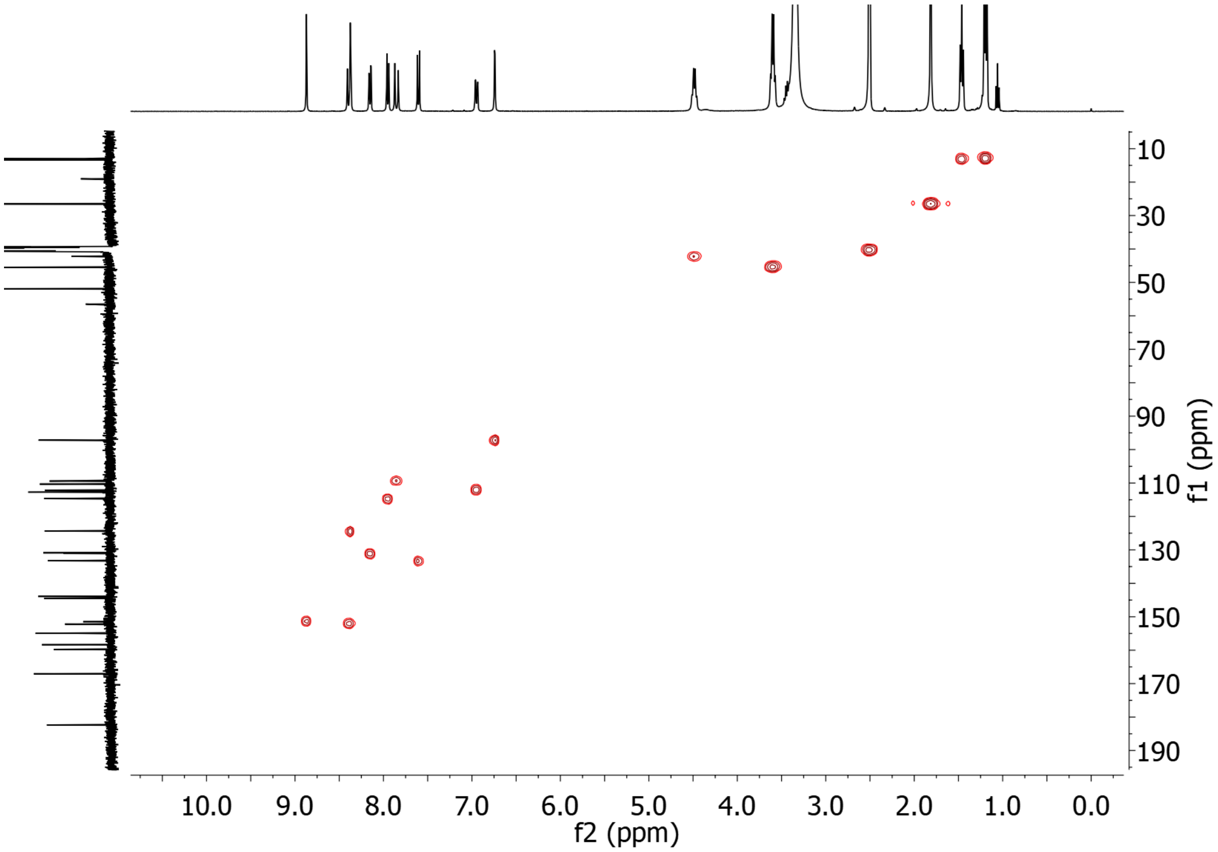

Figure S9. HSQC spectrum of Mito 2 in DMSO-d_6.

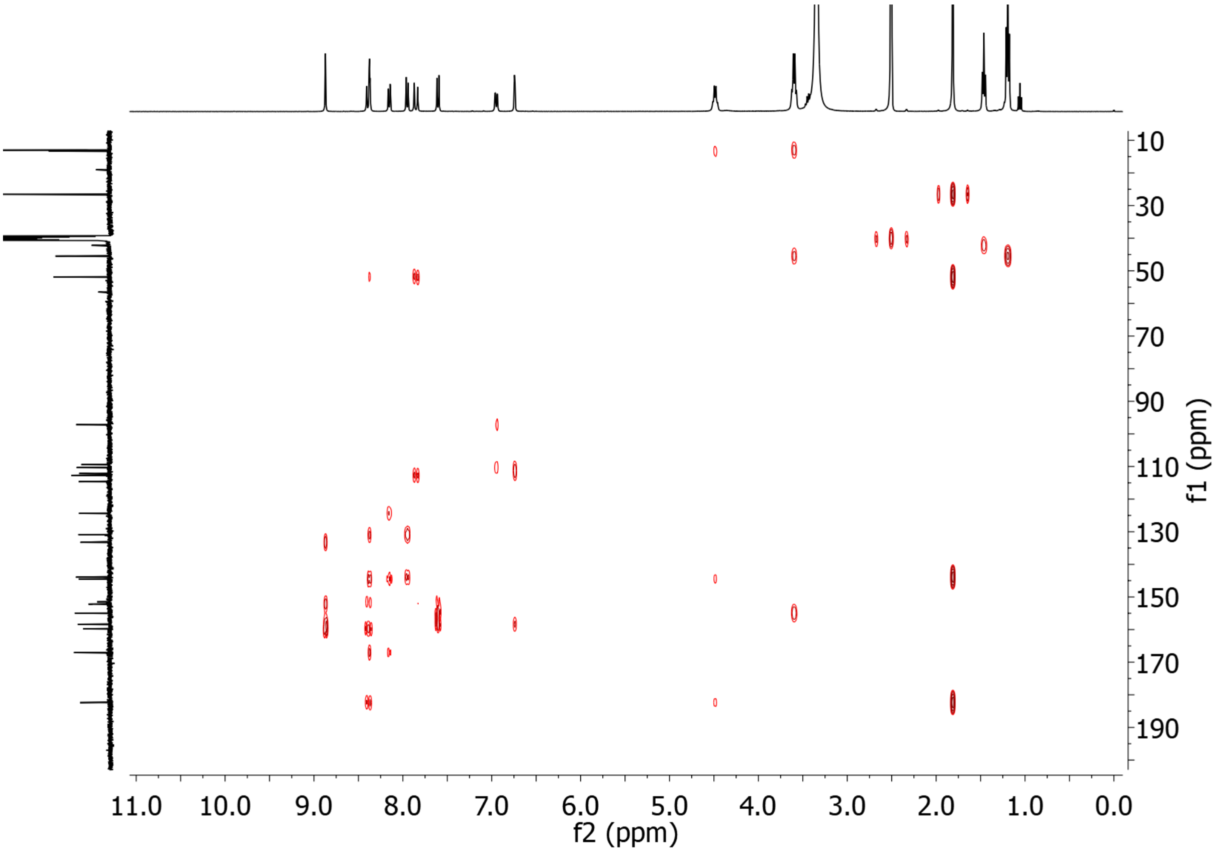

Figure S10. HMBC spectrum of Mito 2 in DMSO-d_6.

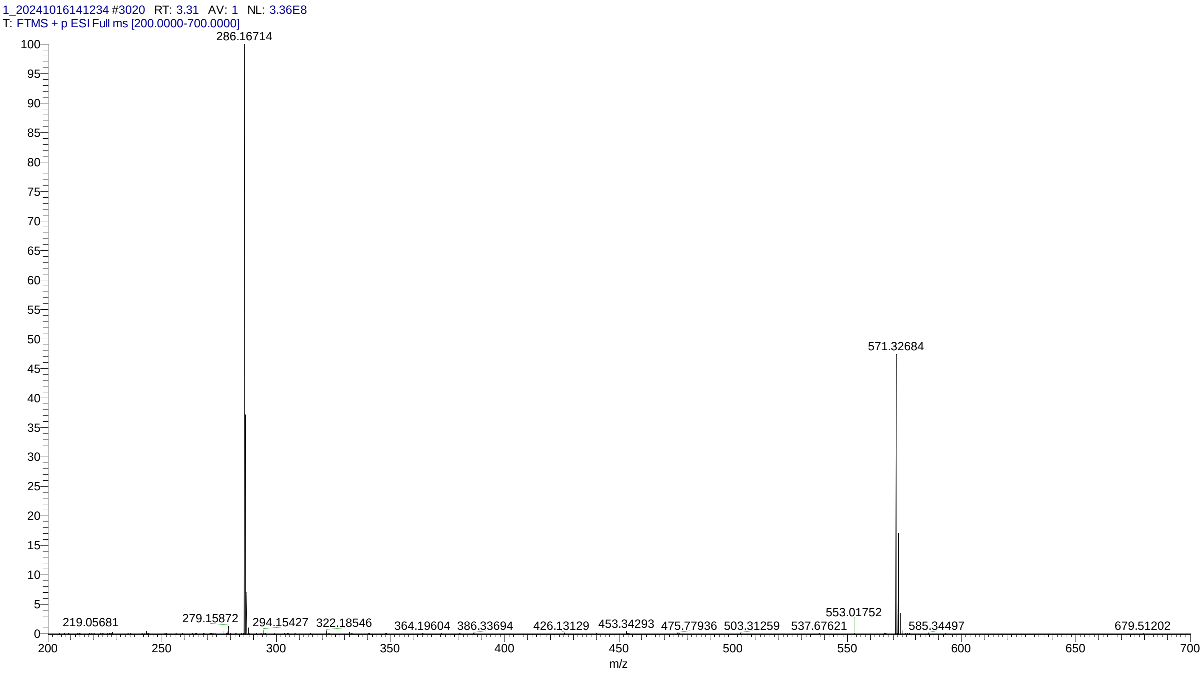

Figure S11. HR-Mass spectrum of **MitoSC**.

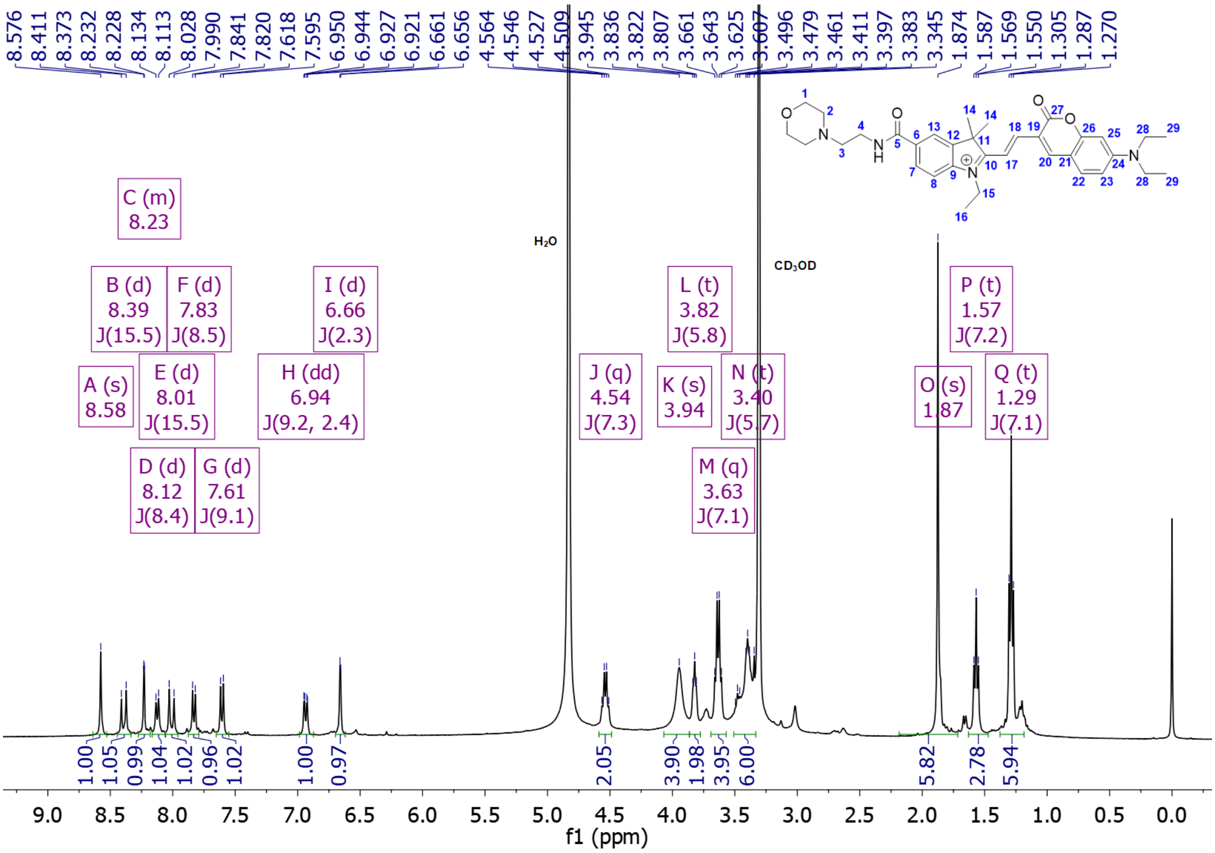

Figure S12. ^{1}H-NMR spectrum of **MitoSC** in CD_3OD. ^{1}H NMR (400 MHz, Methanol-d_4) δ 8.58 (s, 1H, H20), 8.39 (d, J = 15.5 Hz, 1H, H18), 8.23 (s, 1H, H13), 8.12 (d, J = 8.4 Hz, 1H, H7), 8.01 (d, J = 15.5 Hz, 1H, H17), 7.83 (d, J = 8.5 Hz, 1H, H8), 7.61 (d, J = 9.1 Hz, 1H, H22), 6.94 (dd, J = 9.2, 2.4 Hz, 1H, H23), 6.66 (d, J = 2.3 H, 1H, H25), 4.54 (q, J = 7.3 Hz, 2H, H15), 3.94 (br s, 4H, H1), 3.82 (t, J = 5.8 Hz, 2H, H4), 3.63 (q, J = 7.1 Hz, 4H, H28), 3.50-3.35 (m, 6H, H2 and H3), 1.87 (s, 6H, H14), 1.57 (t, J = 7.2 Hz, 3H, H16), 1.29 (t, J = 7.1 Hz, 6H, H29).

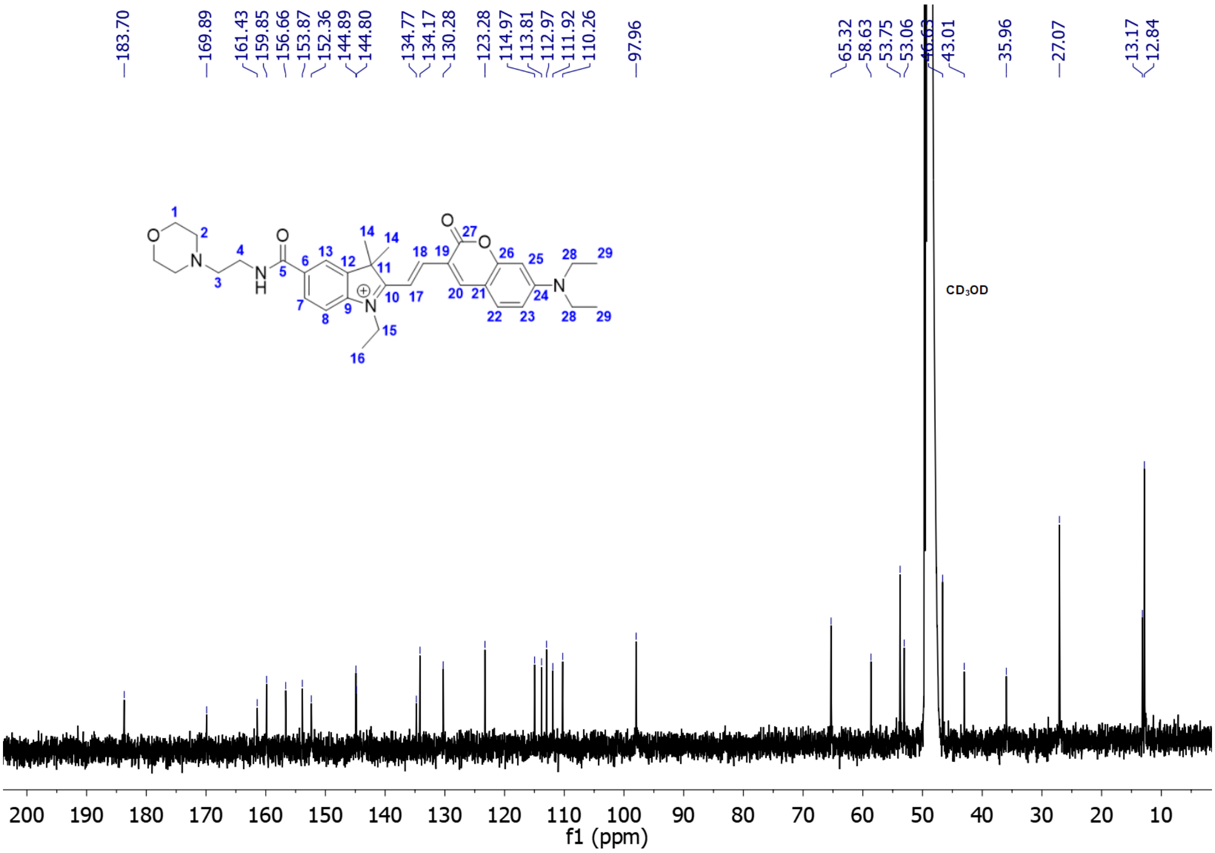

Figure S13. ^{13}C-NMR spectrum of **MitoSC** in CD_3OD. ^{13}C NMR (101 MHz, Methanol-d_4) δ 183.7 (C10), 169.9 (C5), 161.4 (C27), 159.9 (C26), 156.7 (C24), 153.9 (C18), 152.4 (C20), 144.9 (C9/C12), 144.8 (C9/C12), 134.8 (C6), 134.2 (C22), 130.3 (C7), 123.3 (C13), 115.0 (C8), 113.8 (C19), 113.0 (C23), 111.9 (C21), 110.3 (C17), 98.0 (C25), 65.3 (C1), 58.6 (C3), 53.8 (C2), 53.1 (C11), 46.6 (C28), 43.0 (C15), 36.0 (C4), 27.1 (C14), 13.2 (C16), 12.8 (C29).

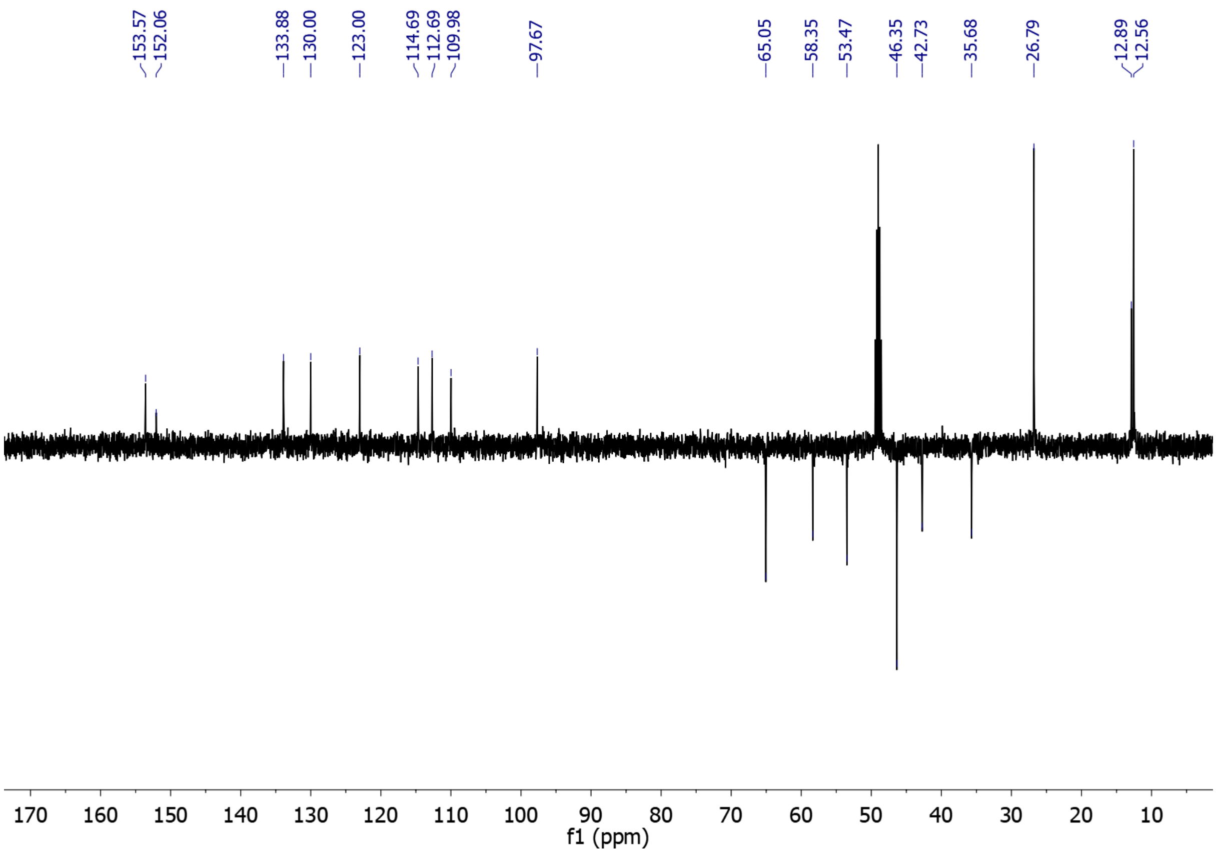

Figure S14. DEPT135 spectrum of **MitoSC** in CD_3OD.

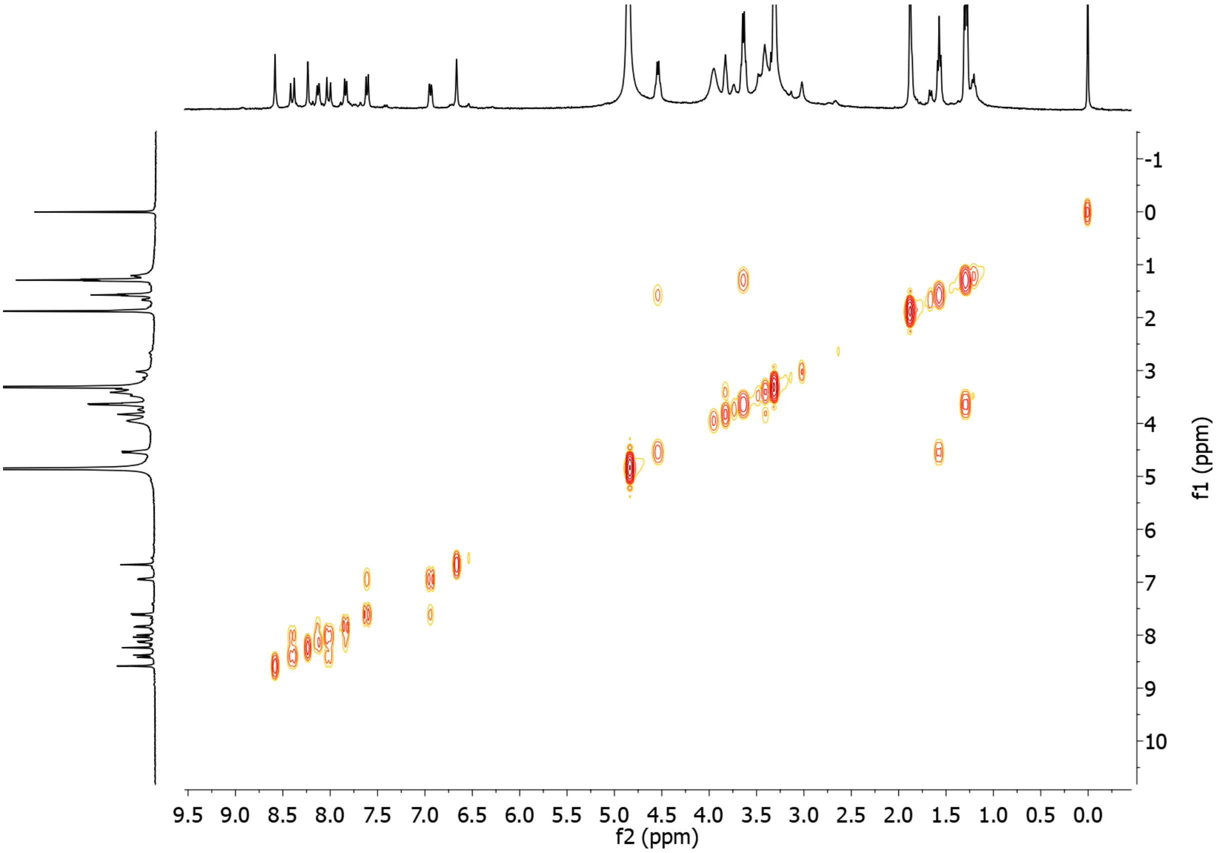

Figure S15. COSY spectrum of **MitoSC** in CD_3OD.

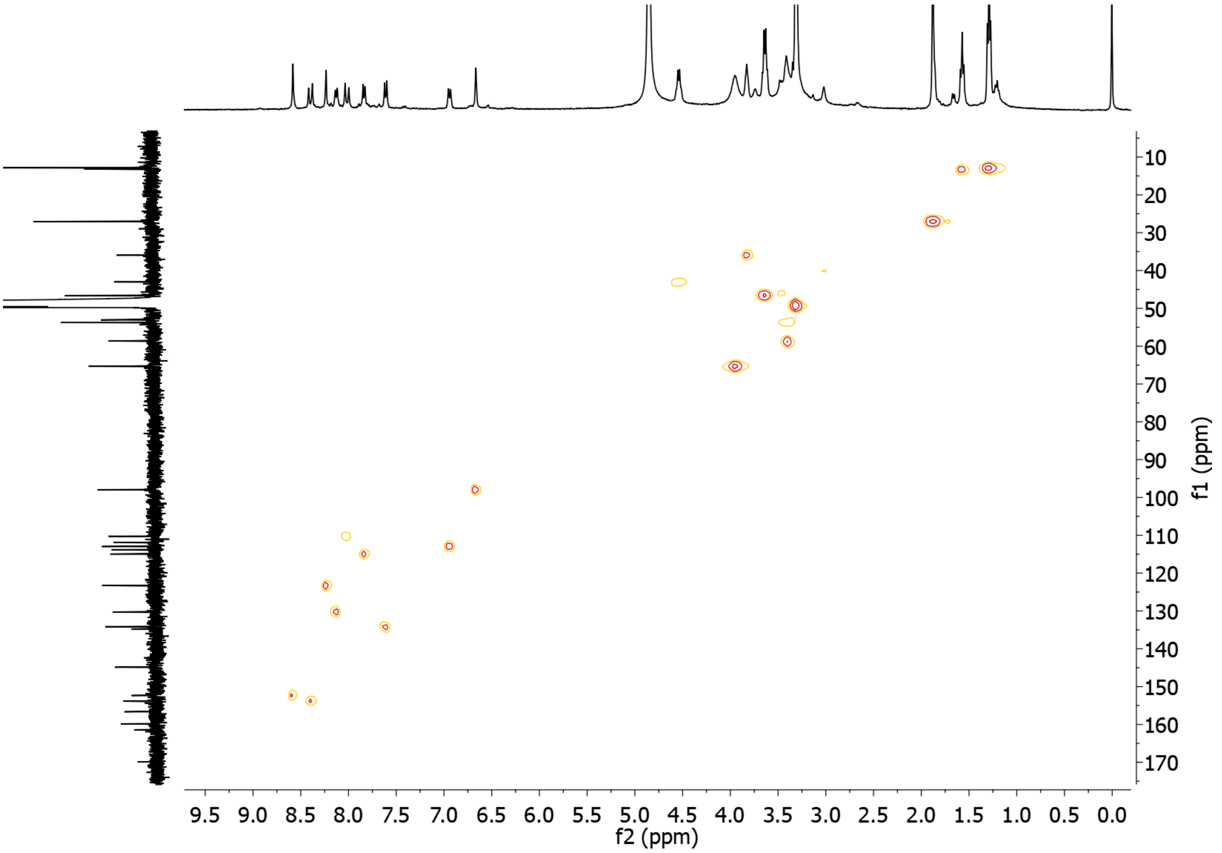

Figure S16. HSQC spectrum of **MitoSC** in CD_3OD.

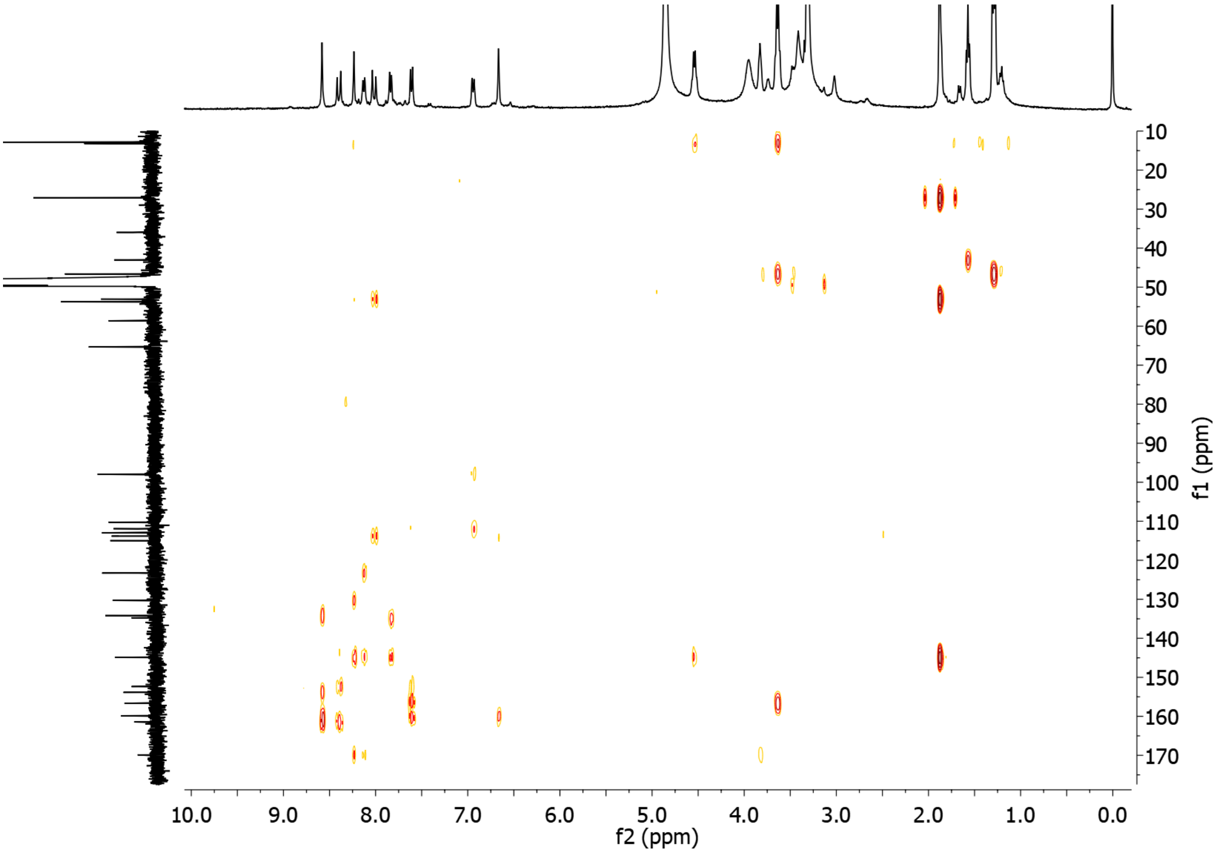

Figure S17. HMBC spectrum of **MitoSC** in CD_3OD.

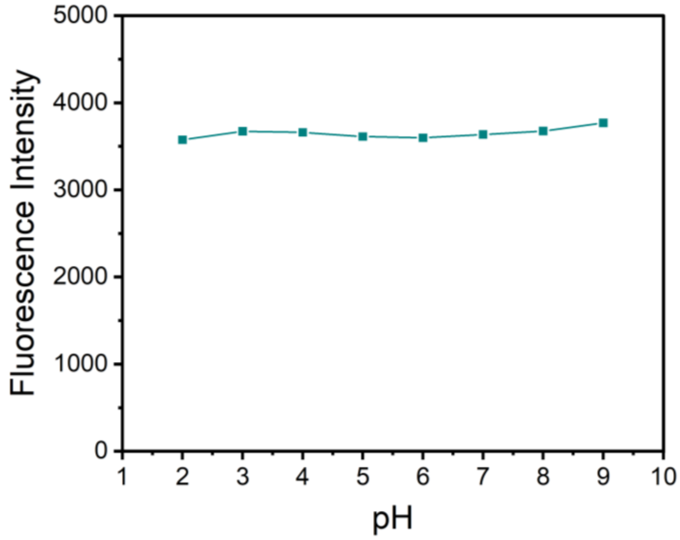

Figure S18. MitoSC exhibits pH stability and remains unaffected by the acidic conditions of lysosomes and the alkaline conditions of mitochondria (λ_{ex} = 561 nm, ex slit: 10 nm, em slit: 10 nm). Measurements were performed 10 min after mixing **MitoSC** with solutions of various pH.

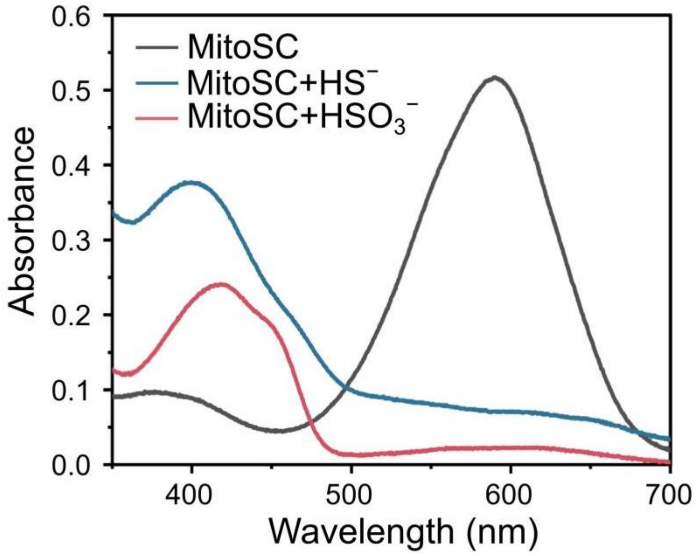

Figure S19. UV-Vis absorption spectra of **MitoSC** (10 μM) with or without HSO_3^-/HS^-(10 μM) in PBS solutions (pH = 7.4) were recorded at room temperature. Measurements were performed 10 min after mixing **MitoSC** and HSO_3^-/HS^-.

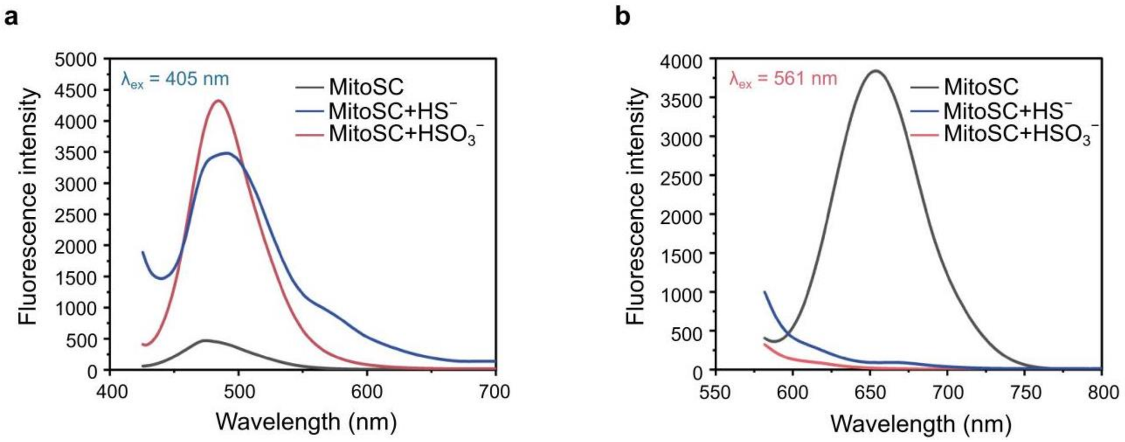

Figure S20. Fluorescence spectra of **MitoSC** before and after reacting with HSO_3^-/HS^-. **(a)** Fluorescence spectra of **MitoSC** (10 μM) with HSO_3^-/HS^- (10 μM) in PBS solutions (pH = 7.4) at room temperature (λ_{ex} = 405 nm, ex slit: 10 nm, em slit: 10 nm). **(b)** Fluorescence spectra of **MitoSC** (10 μM) with HSO_3^-/HS^- (10 μM) in PBS solutions (pH = 7.4) at room temperature (λ_{ex} = 561 nm, ex slit: 10 nm, em slit: 10 nm). Measurements were performed 10 min after mixing **MitoSC** and HSO_3^-/HS^-.

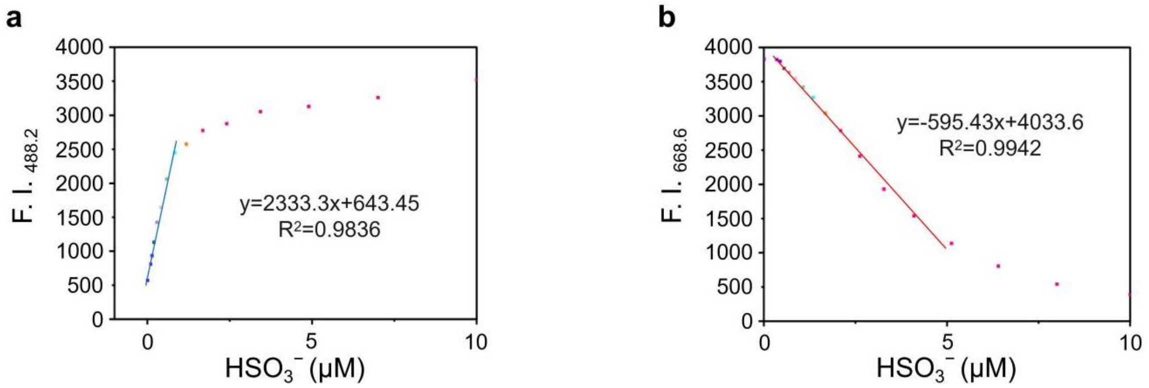

Figure S21. Fluorescence titration experiments of **MitoSC** and HSO_3^-. **(a)** Linear relationship between **MitoSC** and HSO_3^- (0-10 μM) at 488.2 nm (λ_{ex} = 405 nm, ex slit: 10 nm, em slit: 10 nm, R^2 = 0.9836). **(b)** Linear relationship between **MitoSC** and HSO_3^- (0-10 μM) at 668.6 nm (λ_{ex} = 561 nm, ex slit: 10 nm, em slit: 10 nm, R^2 = 0.9942). Measurements were performed 10 min after mixing **MitoSC** and HSO_3^-.

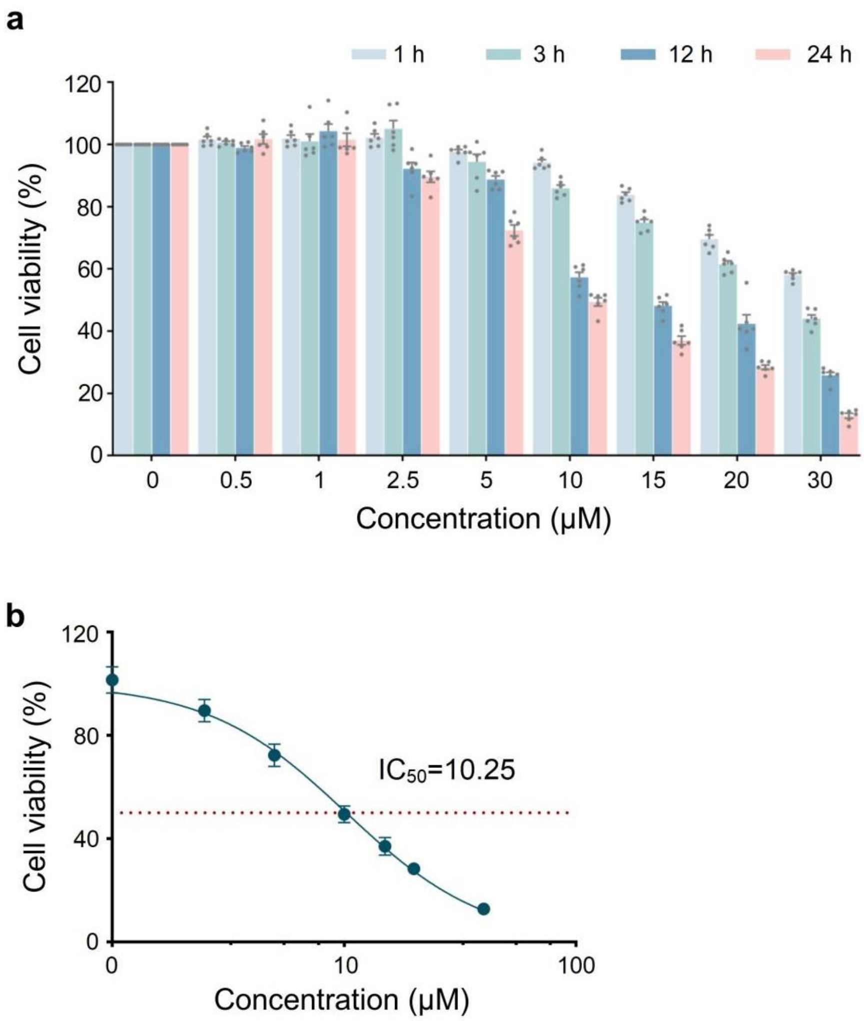

Figure S22. The cytotoxicity assay of **MitoSC**. **(a)** The cytotoxicity of **MitoSC** was assessed at concentrations ranging from 0.5 to 30 μM in HeLa cells (n = 6). **(b)** The dose-response curve and IC_{50} value were derived from the calculation of cell viability (24 h) of **MitoSC** at concentrations of 0.5 to 30 μM in HeLa cells (n = 6). Quantitative data are expressed as mean ± SEM.

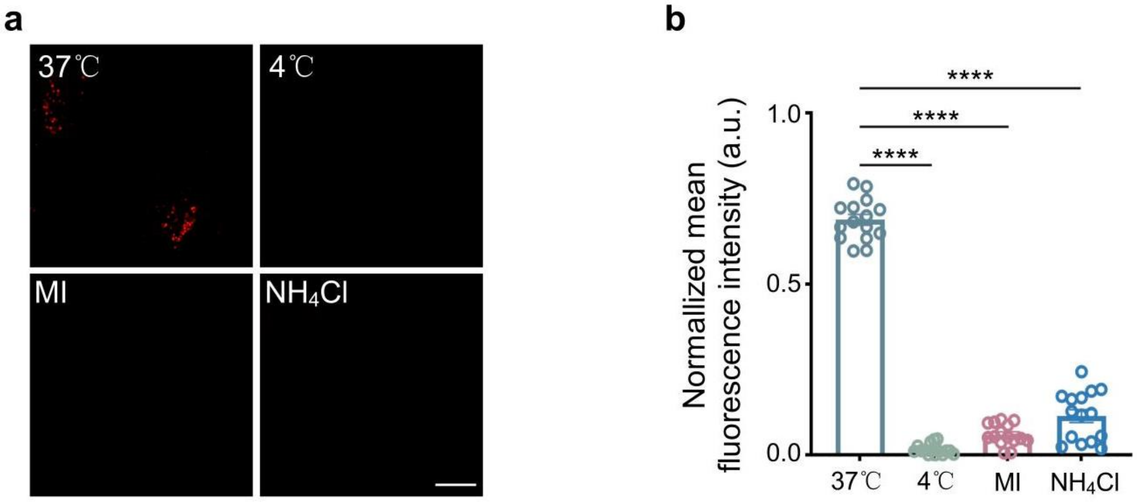

Figure S23. The cell uptake assay of **MitoSC** in HeLa cells. **(a)** Representative images of **MitoSC**-labeled lysosomes at 37℃, and 4℃, as well as metabolic inhibitors (MI) or NH_4Cl (561 nm channel) are shown (scale bar = 10 μm). **(b)** The normalized fluorescence intensity of **MitoSC** in HeLa cells under various treatments (n = 15). Quantitative data are expressed as the mean ± SEM (ns, not significant, *$p < 0.05$, **$p < 0.01$, ***$p < 0.001$, ****$p < 0.0001$).

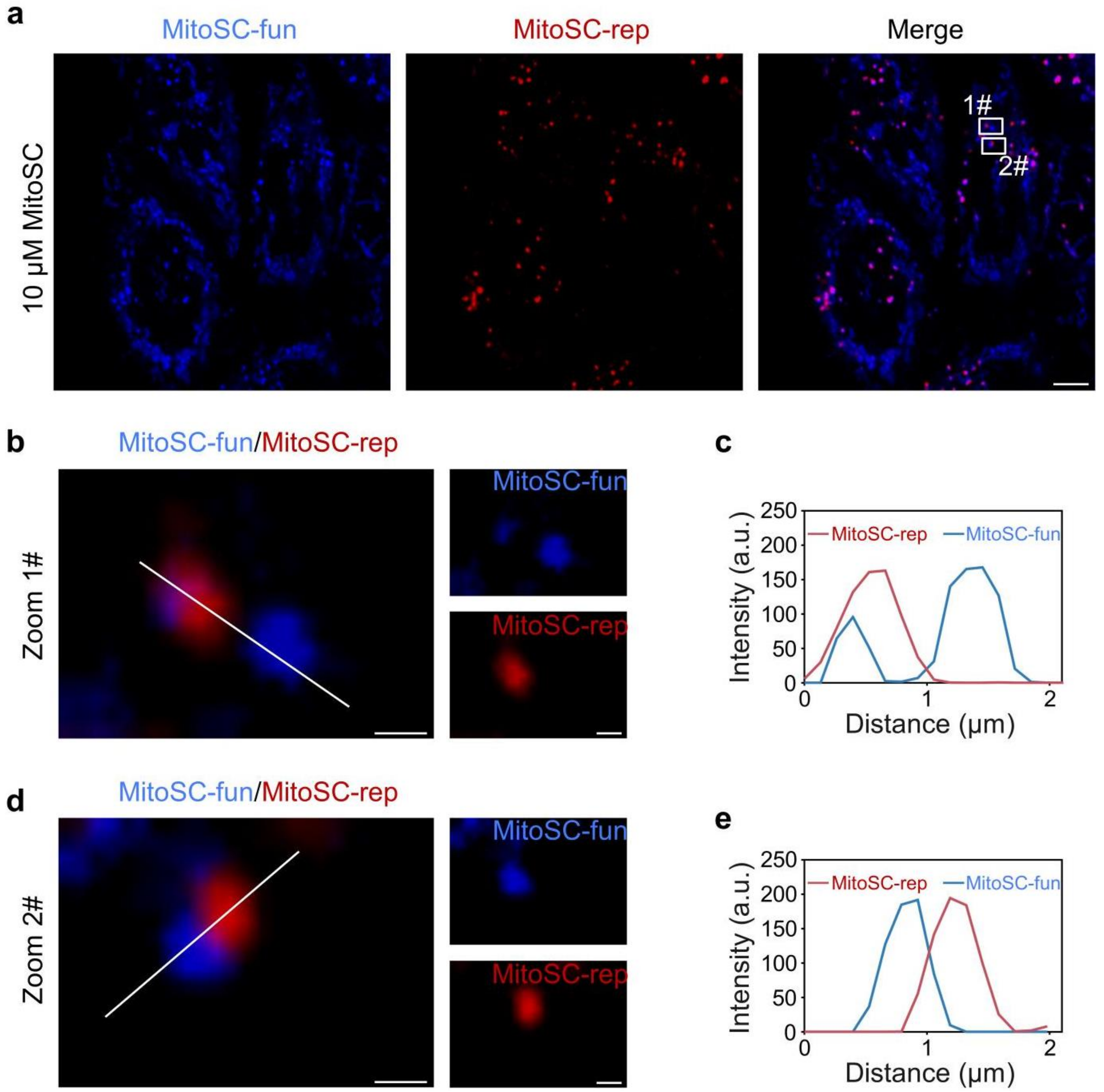

Figure S24. Confocal images of HeLa cells stained with **MitoSC** indicating distinct organelle localization. **(a)** Confocal imaging of HeLa cells stained with **MitoSC** (10 μM) for 30 min (scale bar = 5 μm). **(b)** The white rectangular box 1# in (a) is shown at enlarged scale (scale bar = 0.5 μm). **(c)** Fluorescence intensity distribution of **MitoSC-fun** and **MitoSC-rep** at the position indicated by the white solid line in (b). **(d)** The white rectangular box 2# in (a) is presented at an enlarged scale (scale bar = 0.5 μm). **(e)** Fluorescence intensity distribution of **MitoSC-fun** and **MitoSC-rep** at the position indicated by the white solid line in (d).

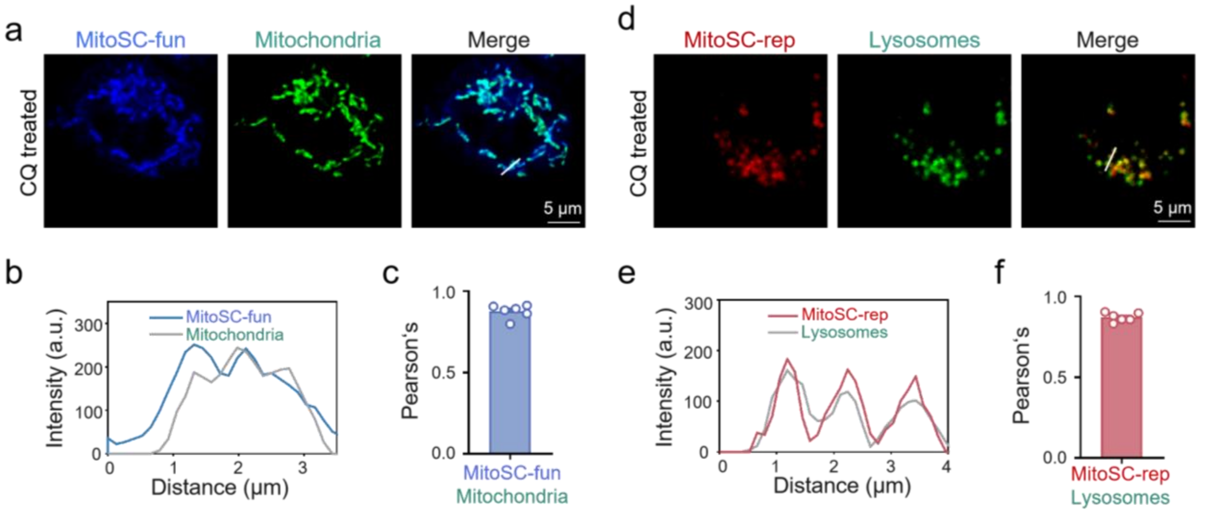

Figure S25. (a) Confocal imaging of HeLa cells co-stained with **MitoSC** (10 μM) and commercial mitochondrial dye (MitoTracker™ Green FM, MTG), which were treated with chloroquine (CQ) (100 μM). **(b)** Fluorescence intensity distribution of **MitoSC-fun** and mitochondria along the position indicated by the white solid line in (a). **(c)** The Pearson correlation coefficient for **MitoSC-fun** with mitochondria is 0.88 (n = 6). **(d)** Confocal imaging of HeLa cells co-stained with **MitoSC** (10 μM) and commercial lysosome dye (LysoTracker™ Green DND-26, LTG) (scale bar = 5 μm), which were treated with chloroquine (CQ) (100 μM). **(e)** Fluorescence intensity distribution of **MitoSC-rep** and lysosomes along the position indicated by the white solid line in (d). **(f)** The Pearson correlation coefficient for **MitoSC-rep** with lysosomes is 0.87 (n = 6). **MitoSC-fun** channel: ex = 405 nm, em = 420-495 nm; **MitoSC-rep** channel: ex = 561 nm, em = 600-640 nm; Mitochondria channel (MTG): ex = 488 nm, em = 500-550 nm; Lysosomes channel (LTG): ex = 488 nm, em = 500-550 nm. Quantitative data are expressed as the mean ± SEM.

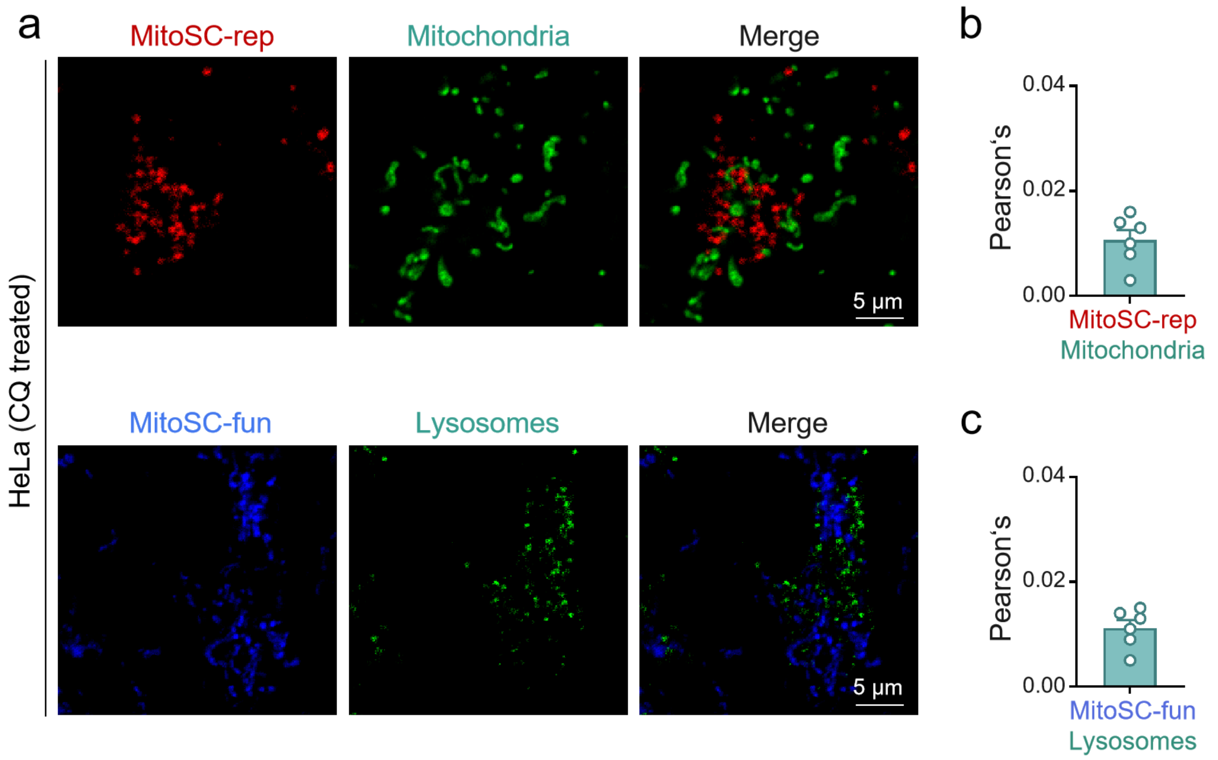

Figure S26. (a) Confocal imaging of HeLa cells co-stained with **MitoSC** (10 μM) and commercial mitochondrial dye (MitoTracker™ Green FM, MTG), and co-stained with **MitoSC** (10 μM) and commercial lysosome dye (LysoTracker™ Green DND-26, LTG). All cells were treated with chloroquine (CQ) (100 μM) (scale bar = 5 μm). **(b)** The Pearson correlation coefficient for **MitoSC-rep** with mitochondria was 0.01 (n = 6). **(c)** The Pearson correlation coefficient for **MitoSC-fun** with lysosomes was 0.01 (n = 6). **MitoSC-fun** channel: ex = 405 nm, em = 420-495 nm; **MitoSC-rep** channel: ex = 561 nm, em = 600-640 nm; Mitochondria channel (MTG): ex = 488 nm, em = 500-550 nm; Lysosomes channel (LTG): ex = 488 nm, em = 500-550 nm. Quantitative data are expressed as the mean ± SEM.

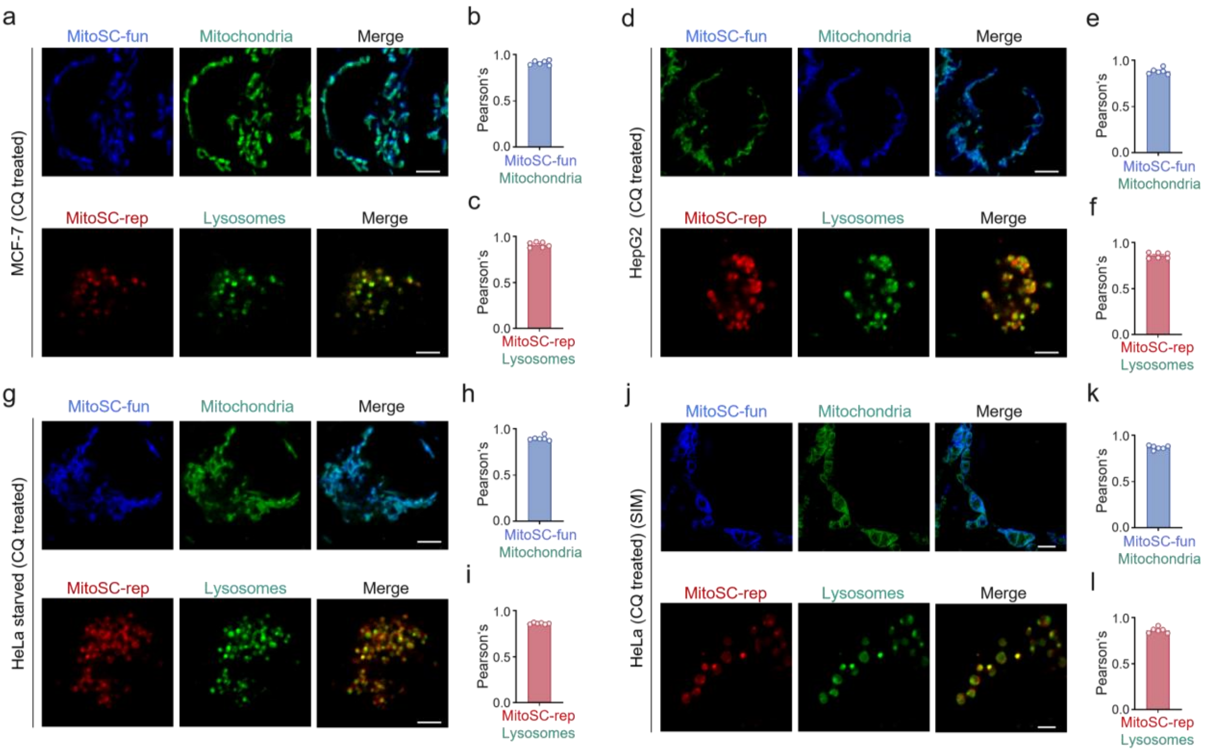

Figure S27. Confocal imaging of **(a)** MCF-7 **(d)** HepG2 **(g)** starved HeLa cells co-stained with **MitoSC** (10 μM) and commercial mitochondrial dye (MitoTracker™ Green FM, MTG); co-stained with **MitoSC** (10 μM) and commercial lysosome dye (LysoTracker™ Green DND-26, LTG) (scale bar = 5 μm), all of which were treated with chloroquine (CQ) (100 μM). The Pearson correlation coefficient for **MitoSC-fun** with mitochondria in **(b)** MCF-7 (0.91) **(e)** HepG2 (0.88) **(h)** starved Hela cells (0.90) (n = 6). The Pearson correlation coefficient for **MitoSC-rep** with lysosomes in **(c)** MCF-7 (0.91) **(f)** HepG2 (0.86) **(i)** starved Hela cells (0.86) (n = 6). **(j)** SIM imaging of Hela cells co-stained with **MitoSC** (10 μM) and commercial mitochondrial dye (MitoTracker™ Green FM, MTG); co-stained with **MitoSC** (10 μM) and commercial lysosome dye (LysoTracker™ Green DND-26, LTG) (scale bar = 2 μm), all of which were treated with chloroquine (CQ) (100 μM). **(k)** The Pearson correlation coefficient for **MitoSC-fun** with mitochondria in SIM imaging of Hela cells was 0.87. **(l)** The Pearson correlation coefficient for **MitoSC-rep** with lysosomes in SIM imaging of Hela cells was 0.86. **MitoSC-fun** channel: ex = 405 nm, em = 420-495 nm; **MitoSC-rep** channel: ex = 561 nm, em = 600-640 nm; Mitochondria channel (MTG): ex = 488 nm, em = 500-550 nm; Lysosomes channel (LTG): ex = 488 nm, em = 500-550 nm. Quantitative data are expressed as the mean ± SEM.

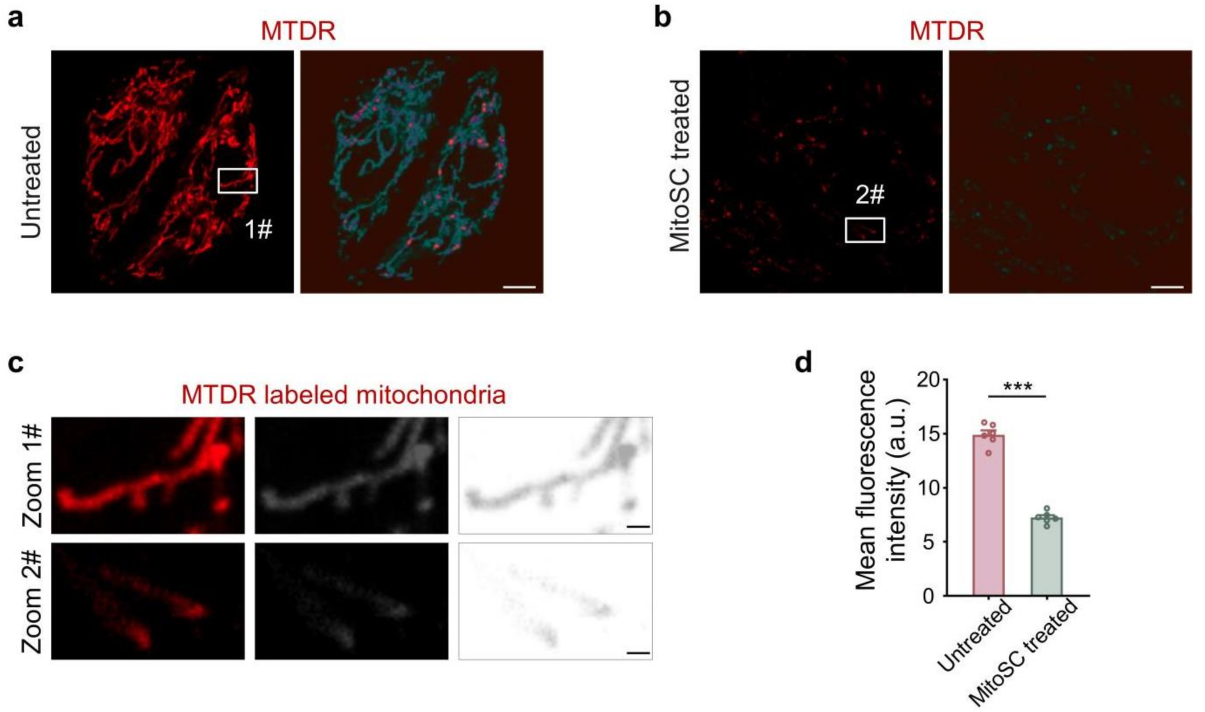

Figure S28. The effect of **MitoSC** treatment on the labeling of mitochondria with MTDR. **(a)** Representative fluorescence images of MTDR-labeled mitochondria without **MitoSC** treatment, the right-side fluorescence image is processed with a Dawn filter, with green to red indicating fluorescence enhancement (scale bar = 5 μm). **(b)** Representative fluorescence images of MTDR-labeled mitochondria with **MitoSC** (10 μM) treatment, the right-side fluorescent image is processed with a Dawn filter, with green to red indicating fluorescence enhancement (scale bar = 5 μm). **(c)** The white rectangular box 1# in (a) and 2# in (b) is presented at an enlarged scale (scale bar = 1 μm). **(d)** Mean fluorescence intensity of MTDR-labeled mitochondria in HeLa cells with or without **MitoSC** treatment (n = 6). Quantitative data are expressed as the mean ± SEM (ns, not significant, **p* < 0.05, ***p* < 0.01, ****p* < 0.001, *****p* < 0.0001).

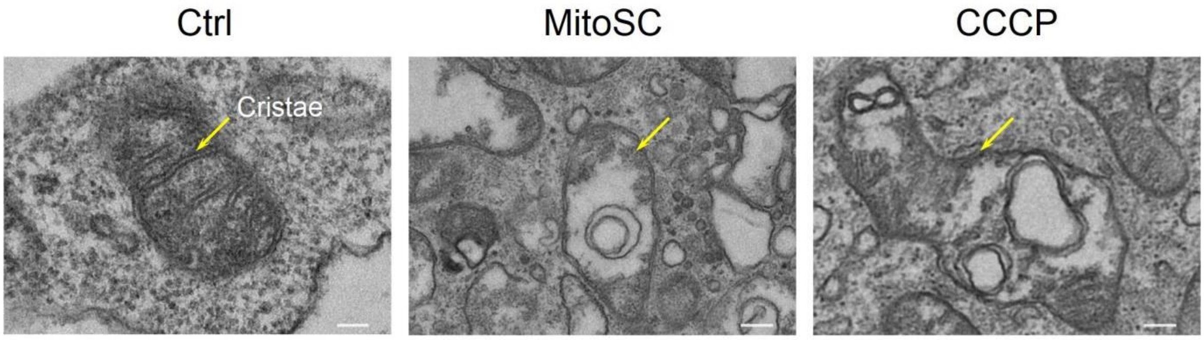

Figure S29. Representative transmission electron microscope images of mitochondria under different treatments are presented, with Ctrl group scale bar at 100 nm, and the **MitoSC** and CCCP groups scale bar at 200 nm.

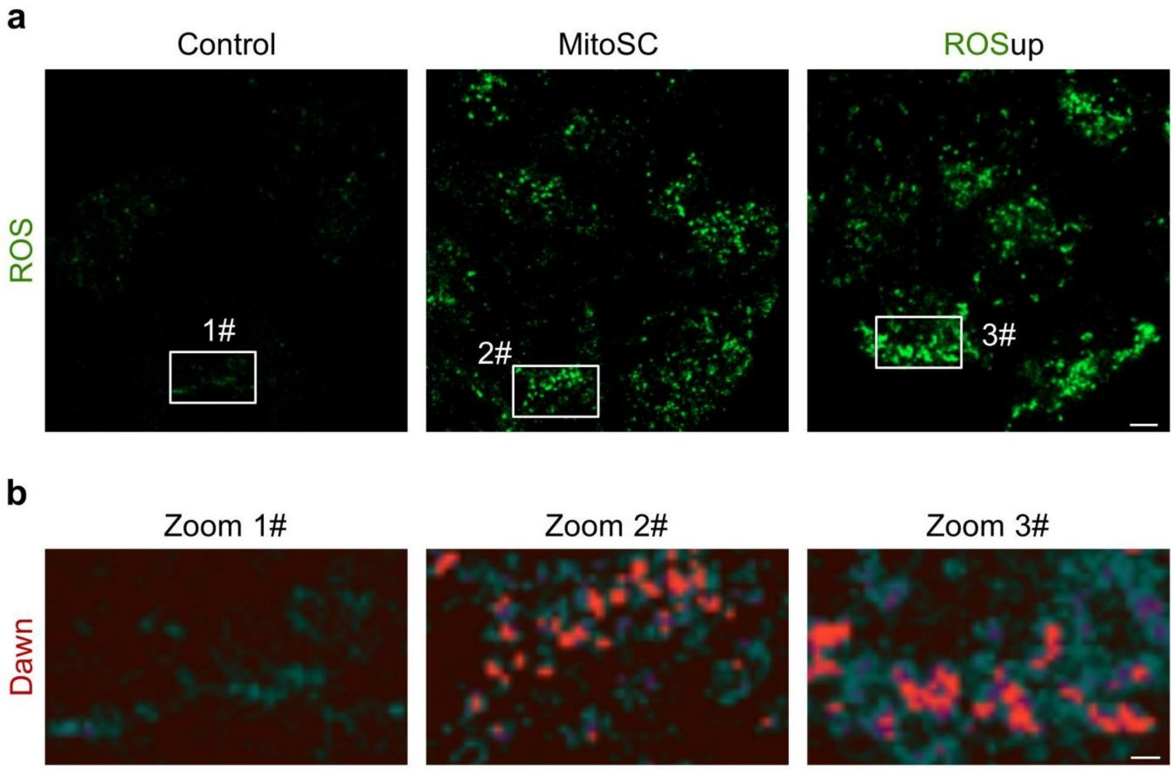

Figure S30. Detection of reactive oxygen species (ROS) levels in HeLa cells treated with 10 μM **MitoSC**. **(a)** Representative fluorescence images of HeLa cells indicating reactive oxygen species (ROS) levels with different treatments. Control group: incubated with 10 μM DCFH-DA for 20 min; **MitoSC** group: first treated with 10 μM **MitoSC** for 30 min, and then incubated with 10 μM DCFH-DA for 20 min; ROSup group: first treated with 10 μM ROSup (S0033S-2, Beyotime) for 30 min, and then incubated with 10 μM DCFH-DA for 20 min (scale bar = 20 μm). **(b)** The white rectangular box 1#, 2# and 3# in (a) was enlarged and these images are processed with a filter (Dawn), green to red indicating fluorescence enhancement (scale bar = 5 μm).

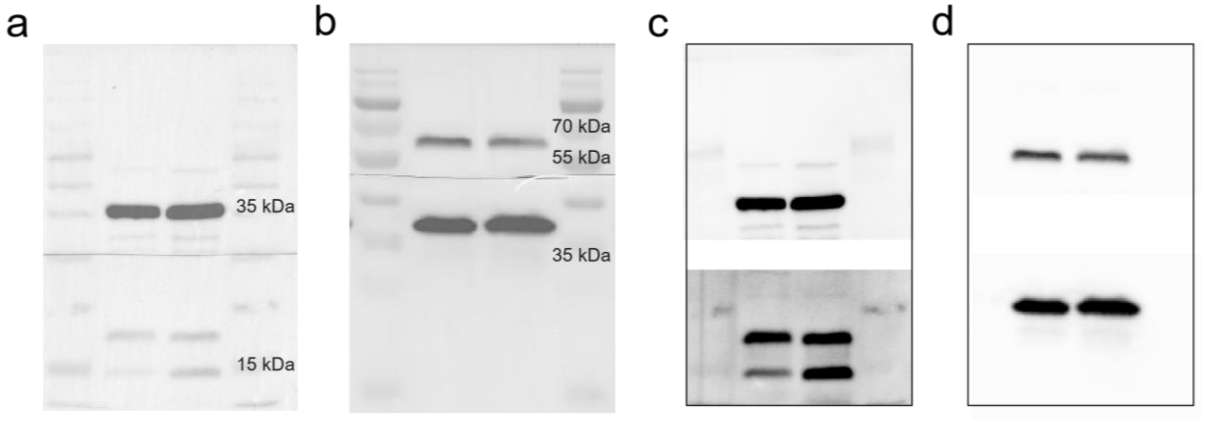

Figure S31. Uncropped blot for figure 3j. **(a)** Merged image of marker, target protein LC3 and reference protein GAPDH. **(b)** Merged image of marker, target protein p62 and reference protein GAPDH. The detection of proteins together can affect their presentation. Therefore, the target protein and reference protein are detected separately **(c)** LC3 and GAPDH **(d)** p62 and GAPDH.

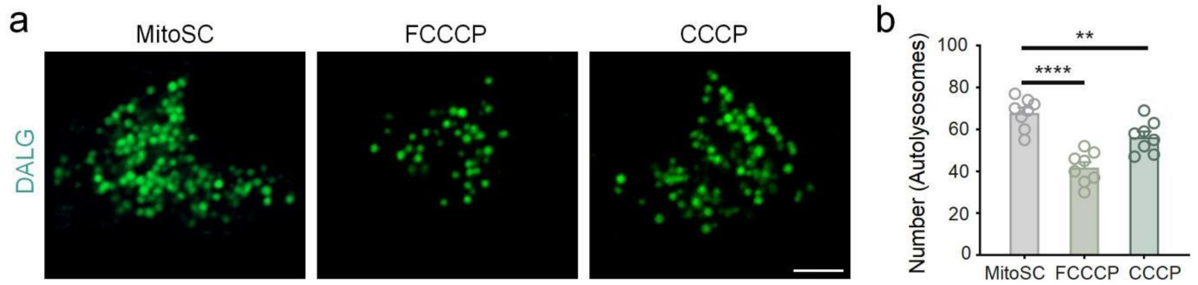

Figure S32. (a) Confocal imaging of HeLa cells treated by **MitoSC**, carbonyl cyanide 4-(trifluoromethoxy)phenylhydrazone (FCCP) and carbonyl cyanide 3-chlorophenylhydrazone (CCCP) and stained with the commercial autolysosome dye DALGreen-Autophagy Detection (DALG) (scale bar = 5 μm). **(b)** The number of autolysosomes in different treatment (n = 8). Autolysosome channel (DALG): ex = 488 nm, em = 500-550 nm. Quantitative data are expressed as the mean ± SEM (ns, not significant; **p* < 0.05, ***p* < 0.01, ****p* < 0.001, *****p* < 0.0001).

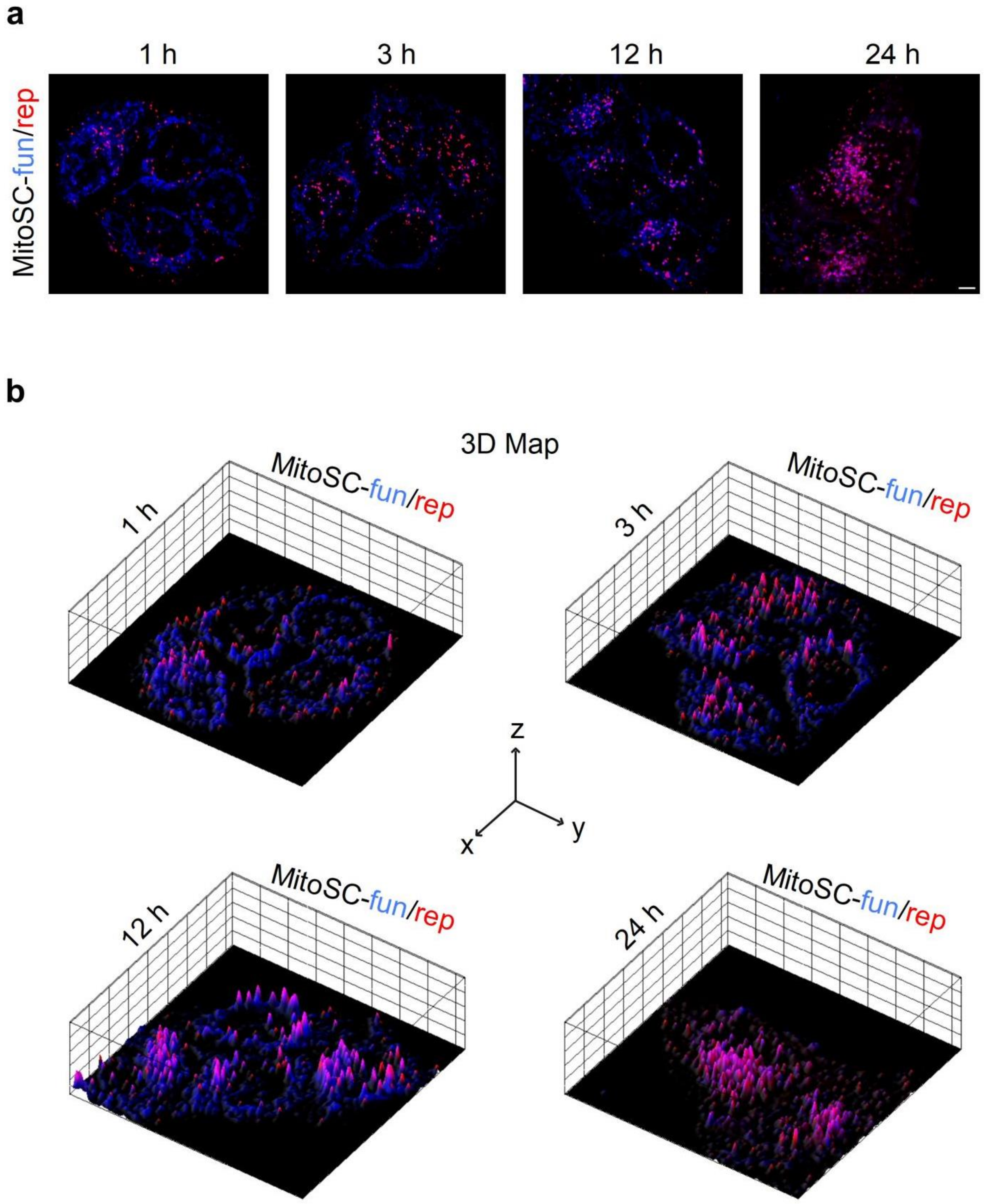

Figure S33. The relationship between the incubation time of **MitoSC** and the level of mitophagy. **(a)** Representative fluorescence images of HeLa cells incubated with **MitoSC** (10 μM) for 1, 3, 12 and 24 h (scale bar = 5 μm). **(b)** Different degrees of overlap between blue (**MitoSC-fun**) and red (**MitoSC-rep**) fluorescence are displayed on a 3D map (Representing different levels of mitophagy).

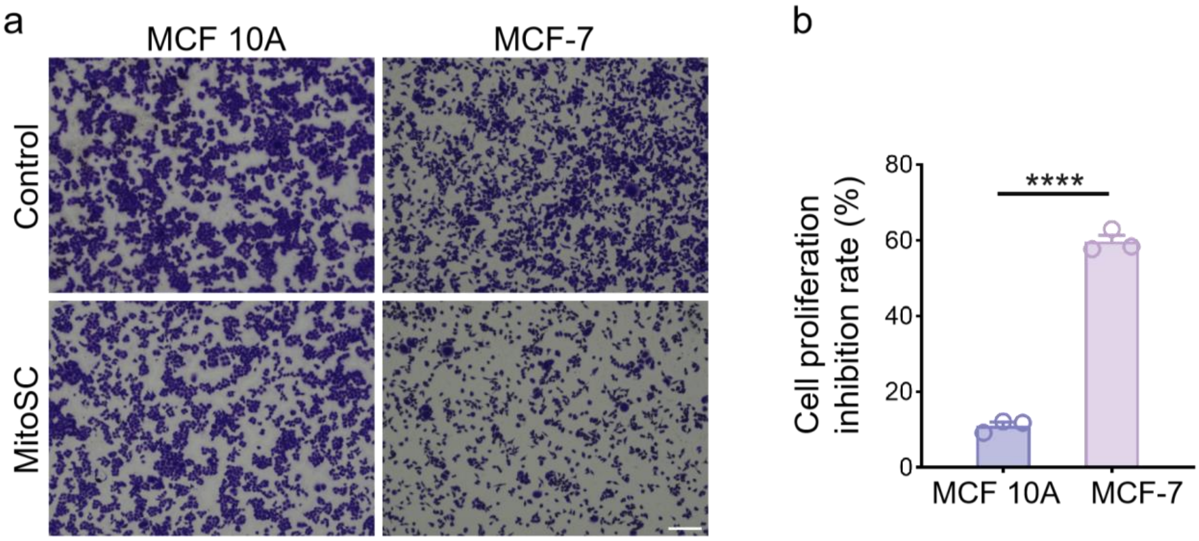

Figure S34. (a) Representative images of crystal violet staining of MCF-7 (breast cancer cells) and MCF 10A (breast epithelial cells) after **MitoSC** treatment (24 h) (scale bar = 200 μm). **(b)** Cell proliferation inhibition rate of **MitoSC** on MCF-7 and MCF 10A cells (n = 3). Quantitative data are expressed as the mean ± SEM (ns, not significant, *$p < 0.05$, **$p < 0.01$, ***$p < 0.001$, ****$p < 0.0001$).

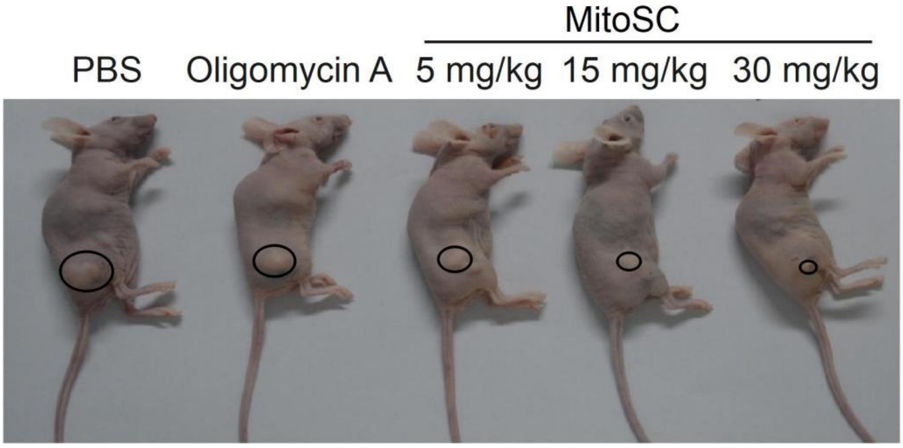

Figure S35. Representative digital photographs of five mouse groups.

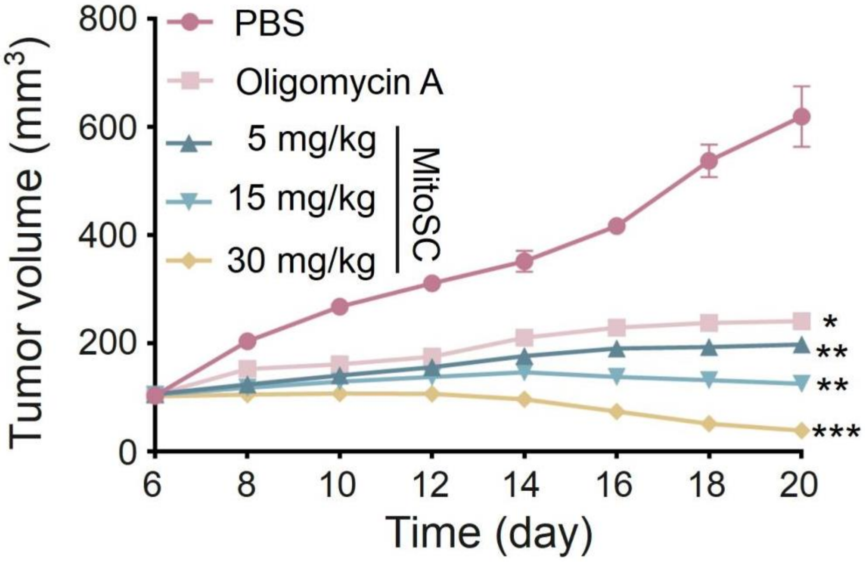

Figure S36. Trend of tumor volume changes in five mouse groups during treatment (n = 5). Quantitative data are expressed as the mean ± SEM (ns, not significant, *$p < 0.05$, **$p < 0.01$, ***$p < 0.001$, ****$p < 0.0001$).

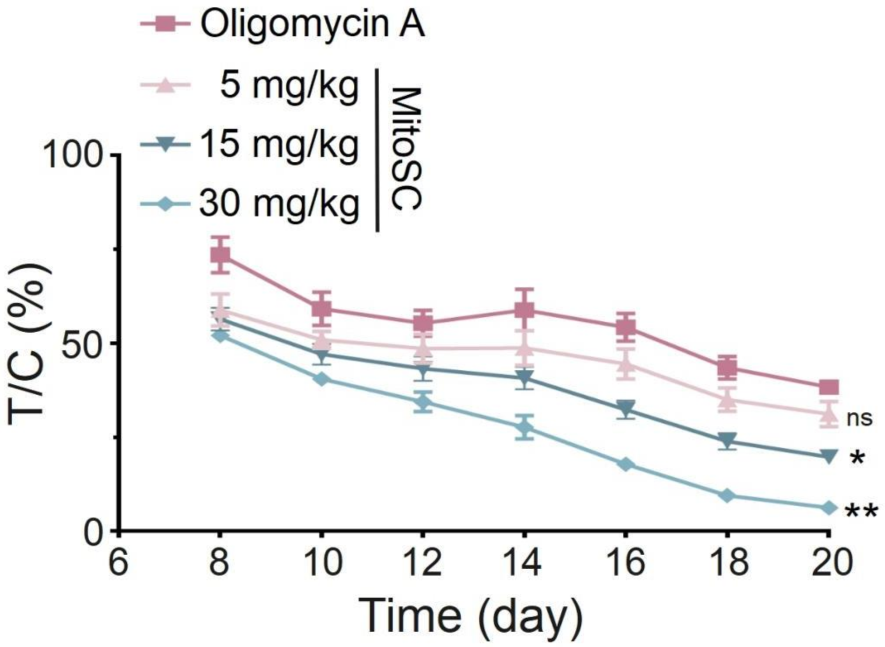

Figure S37. Relative tumor proliferation rate curves for treated groups of mice (n = 5). Quantitative data are expressed as the mean ± SEM (ns, not significant, *$p < 0.05$, **$p < 0.01$, ***$p < 0.001$, ****$p < 0.0001$).

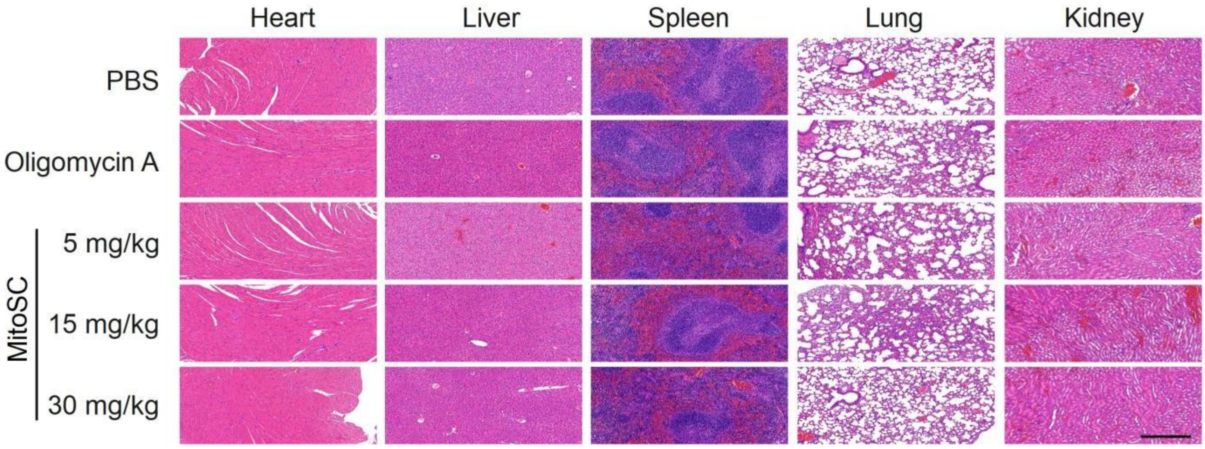

Figure S38. Representative H&E staining images of major tissues (heart, liver, spleen, lung and kidney) after different treatments, revealing that the major tissues were not affected after drug treatments (scale bar = 200 μm).
